# Measurement of the ${{\mathrm{W} }^{+} }\mathrm{W}^{-}$ cross section in pp collisions at $\sqrt{s} = 8$ TeVand limits on anomalous gauge couplings

**DOI:** 10.1140/epjc/s10052-016-4219-1

**Published:** 2016-07-15

**Authors:** V. Khachatryan, A. M. Sirunyan, A. Tumasyan, W. Adam, E. Asilar, T. Bergauer, J. Brandstetter, E. Brondolin, M. Dragicevic, J. Erö, M. Flechl, M. Friedl, R. Frühwirth, V. M. Ghete, C. Hartl, N. Hörmann, J. Hrubec, M. Jeitler, V. Knünz, A. König, M. Krammer, I. Krätschmer, D. Liko, T. Matsushita, I. Mikulec, D. Rabady, B. Rahbaran, H. Rohringer, J. Schieck, R. Schöfbeck, J. Strauss, W. Treberer-Treberspurg, W. Waltenberger, C.-E. Wulz, V. Mossolov, N. Shumeiko, J. Suarez Gonzalez, S. Alderweireldt, T. Cornelis, E. A. De Wolf, X. Janssen, A. Knutsson, J. Lauwers, S. Luyckx, S. Ochesanu, R. Rougny, M. Van De Klundert, H. Van Haevermaet, P. Van Mechelen, N. Van Remortel, A. Van Spilbeeck, S. Abu Zeid, F. Blekman, J. D’Hondt, N. Daci, I. De Bruyn, K. Deroover, N. Heracleous, J. Keaveney, S. Lowette, L. Moreels, A. Olbrechts, Q. Python, D. Strom, S. Tavernier, W. Van Doninck, P. Van Mulders, G. P. Van Onsem, I. Van Parijs, P. Barria, C. Caillol, B. Clerbaux, G. De Lentdecker, H. Delannoy, G. Fasanella, L. Favart, A. P. R. Gay, A. Grebenyuk, T. Lenzi, A. Léonard, T. Maerschalk, A. Marinov, L. Perniè, A. Randle-conde, T. Reis, T. Seva, C. Vander Velde, P. Vanlaer, R. Yonamine, F. Zenoni, F. Zhang, K. Beernaert, L. Benucci, A. Cimmino, S. Crucy, D. Dobur, A. Fagot, G. Garcia, M. Gul, J. Mccartin, A. A. Ocampo Rios, D. Poyraz, D. Ryckbosch, S. Salva, M. Sigamani, N. Strobbe, M. Tytgat, W. Van Driessche, E. Yazgan, N. Zaganidis, S. Basegmez, C. Beluffi, O. Bondu, S. Brochet, G. Bruno, R. Castello, A. Caudron, L. Ceard, G. G. Da Silveira, C. Delaere, D. Favart, L. Forthomme, A. Giammanco, J. Hollar, A. Jafari, P. Jez, M. Komm, V. Lemaitre, A. Mertens, C. Nuttens, L. Perrini, A. Pin, K. Piotrzkowski, A. Popov, L. Quertenmont, M. Selvaggi, M. Vidal Marono, N. Beliy, G. H. Hammad, W. L. Aldá Júnior, G. A. Alves, L. Brito, M. Correa Martins Junior, C. Hensel, C. Mora Herrera, A. Moraes, M. E. Pol, P. Rebello Teles, E. Belchior Batista Das Chagas, W. Carvalho, J. Chinellato, A. Custódio, E. M. Da Costa, D. De Jesus Damiao, C. De Oliveira Martins, S. Fonseca De Souza, L. M. Huertas Guativa, H. Malbouisson, D. Matos Figueiredo, L. Mundim, H. Nogima, W. L. Prado Da Silva, A. Santoro, A. Sznajder, E. J. Tonelli Manganote, A. Vilela Pereira, S. Ahuja, C. A. Bernardes, A. De Souza Santos, S. Dogra, T. R. Fernandez Perez Tomei, E. M. Gregores, P. G. Mercadante, C. S. Moon, S. F. Novaes, Sandra S. Padula, D. Romero Abad, J. C. Ruiz Vargas, A. Aleksandrov, V. Genchev, R. Hadjiiska, P. Iaydjiev, S. Piperov, M. Rodozov, S. Stoykova, G. Sultanov, M. Vutova, A. Dimitrov, I. Glushkov, L. Litov, B. Pavlov, P. Petkov, M. Ahmad, J. G. Bian, G. M. Chen, H. S. Chen, M. Chen, T. Cheng, R. Du, C. H. Jiang, R. Plestina, F. Romeo, S. M. Shaheen, J. Tao, C. Wang, Z. Wang, H. Zhang, C. Asawatangtrakuldee, Y. Ban, Q. Li, S. Liu, Y. Mao, S. J. Qian, D. Wang, Z. Xu, W. Zou, C. Avila, A. Cabrera, L. F. Chaparro Sierra, C. Florez, J. P. Gomez, B. Gomez Moreno, J. C. Sanabria, N. Godinovic, D. Lelas, D. Polic, I. Puljak, P. M. Ribeiro Cipriano, Z. Antunovic, M. Kovac, V. Brigljevic, K. Kadija, J. Luetic, S. Micanovic, L. Sudic, A. Attikis, G. Mavromanolakis, J. Mousa, C. Nicolaou, F. Ptochos, P. A. Razis, H. Rykaczewski, M. Bodlak, M. Finger, M. Finger, A. A. Abdelalim, A. Awad, A. Mahrous, A. Radi, B. Calpas, M. Kadastik, M. Murumaa, M. Raidal, A. Tiko, C. Veelken, P. Eerola, J. Pekkanen, M. Voutilainen, J. Härkönen, V. Karimäki, R. Kinnunen, T. Lampén, K. Lassila-Perini, S. Lehti, T. Lindén, P. Luukka, T. Mäenpää, T. Peltola, E. Tuominen, J. Tuominiemi, E. Tuovinen, L. Wendland, J. Talvitie, T. Tuuva, M. Besancon, F. Couderc, M. Dejardin, D. Denegri, B. Fabbro, J. L. Faure, C. Favaro, F. Ferri, S. Ganjour, A. Givernaud, P. Gras, G. Hamel de Monchenault, P. Jarry, E. Locci, M. Machet, J. Malcles, J. Rander, A. Rosowsky, M. Titov, A. Zghiche, I. Antropov, S. Baffioni, F. Beaudette, P. Busson, L. Cadamuro, E. Chapon, C. Charlot, T. Dahms, O. Davignon, N. Filipovic, A. Florent, R. Granier de Cassagnac, S. Lisniak, L. Mastrolorenzo, P. Miné, I. N. Naranjo, M. Nguyen, C. Ochando, G. Ortona, P. Paganini, S. Regnard, R. Salerno, J. B. Sauvan, Y. Sirois, T. Strebler, Y. Yilmaz, A. Zabi, J.-L. Agram, J. Andrea, A. Aubin, D. Bloch, J.-M. Brom, M. Buttignol, E. C. Chabert, N. Chanon, C. Collard, E. Conte, X. Coubez, J.-C. Fontaine, D. Gelé, U. Goerlach, C. Goetzmann, A.-C. Le Bihan, J. A. Merlin, K. Skovpen, P. Van Hove, S. Gadrat, S. Beauceron, C. Bernet, G. Boudoul, E. Bouvier, C. A. Carrillo Montoya, J. Chasserat, R. Chierici, D. Contardo, B. Courbon, P. Depasse, H. El Mamouni, J. Fan, J. Fay, S. Gascon, M. Gouzevitch, B. Ille, F. Lagarde, I. B. Laktineh, M. Lethuillier, L. Mirabito, A. L. Pequegnot, S. Perries, J. D. Ruiz Alvarez, D. Sabes, L. Sgandurra, V. Sordini, M. Vander Donckt, P. Verdier, S. Viret, H. Xiao, T. Toriashvili, D. Lomidze, C. Autermann, S. Beranek, M. Edelhoff, L. Feld, A. Heister, M. K. Kiesel, K. Klein, M. Lipinski, A. Ostapchuk, M. Preuten, F. Raupach, S. Schael, J. F. Schulte, T. Verlage, H. Weber, B. Wittmer, V. Zhukov, M. Ata, M. Brodski, E. Dietz-Laursonn, D. Duchardt, M. Endres, M. Erdmann, S. Erdweg, T. Esch, R. Fischer, A. Güth, T. Hebbeker, C. Heidemann, K. Hoepfner, D. Klingebiel, S. Knutzen, P. Kreuzer, M. Merschmeyer, A. Meyer, P. Millet, M. Olschewski, K. Padeken, P. Papacz, T. Pook, M. Radziej, H. Reithler, M. Rieger, F. Scheuch, L. Sonnenschein, D. Teyssier, S. Thüer, V. Cherepanov, Y. Erdogan, G. Flügge, H. Geenen, M. Geisler, F. Hoehle, B. Kargoll, T. Kress, Y. Kuessel, A. Künsken, J. Lingemann, A. Nehrkorn, A. Nowack, I. M. Nugent, C. Pistone, O. Pooth, A. Stahl, M. Aldaya Martin, I. Asin, N. Bartosik, O. Behnke, U. Behrens, A. J. Bell, K. Borras, A. Burgmeier, A. Cakir, L. Calligaris, A. Campbell, S. Choudhury, F. Costanza, C. Diez Pardos, G. Dolinska, S. Dooling, T. Dorland, G. Eckerlin, D. Eckstein, T. Eichhorn, G. Flucke, E. Gallo, J. Garay Garcia, A. Geiser, A. Gizhko, P. Gunnellini, J. Hauk, M. Hempel, H. Jung, A. Kalogeropoulos, O. Karacheban, M. Kasemann, P. Katsas, J. Kieseler, C. Kleinwort, I. Korol, W. Lange, J. Leonard, K. Lipka, A. Lobanov, W. Lohmann, R. Mankel, I. Marfin, I.-A. Melzer-Pellmann, A. B. Meyer, G. Mittag, J. Mnich, A. Mussgiller, S. Naumann-Emme, A. Nayak, E. Ntomari, H. Perrey, D. Pitzl, R. Placakyte, A. Raspereza, B. Roland, M. Ö. Sahin, P. Saxena, T. Schoerner-Sadenius, M. Schröder, C. Seitz, S. Spannagel, K. D. Trippkewitz, C. Wissing, V. Blobel, M. Centis Vignali, A. R. Draeger, J. Erfle, E. Garutti, K. Goebel, D. Gonzalez, M. Görner, J. Haller, M. Hoffmann, R. S. Höing, A. Junkes, R. Klanner, R. Kogler, T. Lapsien, T. Lenz, I. Marchesini, D. Marconi, D. Nowatschin, J. Ott, F. Pantaleo, T. Peiffer, A. Perieanu, N. Pietsch, J. Poehlsen, D. Rathjens, C. Sander, H. Schettler, P. Schleper, E. Schlieckau, A. Schmidt, J. Schwandt, M. Seidel, V. Sola, H. Stadie, G. Steinbrück, H. Tholen, D. Troendle, E. Usai, L. Vanelderen, A. Vanhoefer, M. Akbiyik, C. Barth, C. Baus, J. Berger, C. Böser, E. Butz, T. Chwalek, F. Colombo, W. De Boer, A. Descroix, A. Dierlamm, S. Fink, F. Frensch, M. Giffels, A. Gilbert, F. Hartmann, S. M. Heindl, U. Husemann, F. Kassel, I. Katkov, A. Kornmayer, P. Lobelle Pardo, B. Maier, H. Mildner, M. U. Mozer, T. Müller, Th. Müller, M. Plagge, G. Quast, K. Rabbertz, S. Röcker, F. Roscher, H. J. Simonis, F. M. Stober, R. Ulrich, J. Wagner-Kuhr, S. Wayand, M. Weber, T. Weiler, C. Wöhrmann, R. Wolf, G. Anagnostou, G. Daskalakis, T. Geralis, V. A. Giakoumopoulou, A. Kyriakis, D. Loukas, A. Psallidas, I. Topsis-Giotis, A. Agapitos, S. Kesisoglou, A. Panagiotou, N. Saoulidou, E. Tziaferi, I. Evangelou, G. Flouris, C. Foudas, P. Kokkas, N. Loukas, N. Manthos, I. Papadopoulos, E. Paradas, J. Strologas, G. Bencze, C. Hajdu, A. Hazi, P. Hidas, D. Horvath, F. Sikler, V. Veszpremi, G. Vesztergombi, A. J. Zsigmond, N. Beni, S. Czellar, J. Karancsi, J. Molnar, Z. Szillasi, M. Bartók, A. Makovec, P. Raics, Z. L. Trocsanyi, B. Ujvari, P. Mal, K. Mandal, N. Sahoo, S. K. Swain, S. Bansal, S. B. Beri, V. Bhatnagar, R. Chawla, R. Gupta, U. Bhawandeep, A. K. Kalsi, A. Kaur, M. Kaur, R. Kumar, A. Mehta, M. Mittal, N. Nishu, J. B. Singh, G. Walia, Ashok Kumar, Arun Kumar, A. Bhardwaj, B. C. Choudhary, R. B. Garg, A. Kumar, S. Malhotra, M. Naimuddin, K. Ranjan, R. Sharma, V. Sharma, S. Banerjee, S. Bhattacharya, K. Chatterjee, S. Dey, S. Dutta, Sa. Jain, N. Majumdar, A. Modak, K. Mondal, S. Mukherjee, S. Mukhopadhyay, A. Roy, D. Roy, S. Roy Chowdhury, S. Sarkar, M. Sharan, A. Abdulsalam, R. Chudasama, D. Dutta, V. Jha, V. Kumar, A. K. Mohanty, L. M. Pant, P. Shukla, A. Topkar, T. Aziz, S. Banerjee, S. Bhowmik, R. M. Chatterjee, R. K. Dewanjee, S. Dugad, S. Ganguly, S. Ghosh, M. Guchait, A. Gurtu, G. Kole, S. Kumar, B. Mahakud, M. Maity, G. Majumder, K. Mazumdar, S. Mitra, G. B. Mohanty, B. Parida, T. Sarkar, K. Sudhakar, N. Sur, B. Sutar, N. Wickramage, S. Chauhan, S. Dube, S. Sharma, H. Bakhshiansohi, H. Behnamian, S. M. Etesami, A. Fahim, R. Goldouzian, M. Khakzad, M. Mohammadi Najafabadi, M. Naseri, S. Paktinat Mehdiabadi, F. Rezaei Hosseinabadi, B. Safarzadeh, M. Zeinali, M. Felcini, M. Grunewald, M. Abbrescia, C. Calabria, C. Caputo, S. S. Chhibra, A. Colaleo, D. Creanza, L. Cristella, N. De Filippis, M. De Palma, L. Fiore, G. Iaselli, G. Maggi, M. Maggi, G. Miniello, S. My, S. Nuzzo, A. Pompili, G. Pugliese, R. Radogna, A. Ranieri, G. Selvaggi, L. Silvestris, R. Venditti, P. Verwilligen, G. Abbiendi, C. Battilana, A. C. Benvenuti, D. Bonacorsi, S. Braibant-Giacomelli, L. Brigliadori, R. Campanini, P. Capiluppi, A. Castro, F. R. Cavallo, G. Codispoti, M. Cuffiani, G. M. Dallavalle, F. Fabbri, A. Fanfani, D. Fasanella, P. Giacomelli, C. Grandi, L. Guiducci, S. Marcellini, G. Masetti, A. Montanari, F. L. Navarria, A. Perrotta, A. M. Rossi, T. Rovelli, G. P. Siroli, N. Tosi, R. Travaglini, G. Cappello, M. Chiorboli, S. Costa, F. Giordano, R. Potenza, A. Tricomi, C. Tuve, G. Barbagli, V. Ciulli, C. Civinini, R. D’Alessandro, E. Focardi, S. Gonzi, V. Gori, P. Lenzi, M. Meschini, S. Paoletti, G. Sguazzoni, A. Tropiano, L. Viliani, L. Benussi, S. Bianco, F. Fabbri, D. Piccolo, V. Calvelli, F. Ferro, M. Lo Vetere, M. R. Monge, E. Robutti, S. Tosi, L. Brianza, M. E. Dinardo, S. Fiorendi, S. Gennai, R. Gerosa, A. Ghezzi, P. Govoni, S. Malvezzi, R. A. Manzoni, B. Marzocchi, D. Menasce, L. Moroni, M. Paganoni, D. Pedrini, S. Ragazzi, N. Redaelli, T. Tabarelli de Fatis, S. Buontempo, N. Cavallo, S. Di Guida, M. Esposito, F. Fabozzi, A. O. M. Iorio, G. Lanza, L. Lista, S. Meola, M. Merola, P. Paolucci, C. Sciacca, F. Thyssen, P. Azzi, N. Bacchetta, L. Benato, D. Bisello, A. Boletti, R. Carlin, A. Carvalho Antunes De Oliveira, P. Checchia, M. Dall’Osso, T. Dorigo, U. Dosselli, F. Gasparini, U. Gasparini, A. Gozzelino, S. Lacaprara, M. Margoni, A. T. Meneguzzo, J. Pazzini, M. Pegoraro, N. Pozzobon, P. Ronchese, F. Simonetto, E. Torassa, M. Tosi, S. Vanini, M. Zanetti, P. Zotto, A. Zucchetta, G. Zumerle, A. Braghieri, A. Magnani, P. Montagna, S. P. Ratti, V. Re, C. Riccardi, P. Salvini, I. Vai, P. Vitulo, L. Alunni Solestizi, M. Biasini, G. M. Bilei, D. Ciangottini, L. Fanò, P. Lariccia, G. Mantovani, M. Menichelli, A. Saha, A. Santocchia, A. Spiezia, K. Androsov, P. Azzurri, G. Bagliesi, J. Bernardini, T. Boccali, G. Broccolo, R. Castaldi, M. A. Ciocci, R. Dell’Orso, S. Donato, G. Fedi, L. Foà, A. Giassi, M. T. Grippo, F. Ligabue, T. Lomtadze, L. Martini, A. Messineo, F. Palla, A. Rizzi, A. Savoy-Navarro, A. T. Serban, P. Spagnolo, P. Squillacioti, R. Tenchini, G. Tonelli, A. Venturi, P. G. Verdini, L. Barone, F. Cavallari, G. D’imperio, D. Del Re, M. Diemoz, S. Gelli, C. Jorda, E. Longo, F. Margaroli, P. Meridiani, F. Micheli, G. Organtini, R. Paramatti, F. Preiato, S. Rahatlou, C. Rovelli, F. Santanastasio, P. Traczyk, N. Amapane, R. Arcidiacono, S. Argiro, M. Arneodo, R. Bellan, C. Biino, N. Cartiglia, M. Costa, R. Covarelli, A. Degano, N. Demaria, L. Finco, B. Kiani, C. Mariotti, S. Maselli, E. Migliore, V. Monaco, E. Monteil, M. Musich, M. M. Obertino, L. Pacher, N. Pastrone, M. Pelliccioni, G. L. Pinna Angioni, F. Ravera, A. Romero, M. Ruspa, R. Sacchi, A. Solano, A. Staiano, U. Tamponi, S. Belforte, V. Candelise, M. Casarsa, F. Cossutti, G. Della Ricca, B. Gobbo, C. La Licata, M. Marone, A. Schizzi, T. Umer, A. Zanetti, S. Chang, A. Kropivnitskaya, S. K. Nam, D. H. Kim, G. N. Kim, M. S. Kim, D. J. Kong, S. Lee, Y. D. Oh, A. Sakharov, D. C. Son, J. A. Brochero Cifuentes, H. Kim, T. J. Kim, M. S. Ryu, S. Song, S. Choi, Y. Go, D. Gyun, B. Hong, M. Jo, H. Kim, Y. Kim, B. Lee, K. Lee, K. S. Lee, S. Lee, S. K. Park, Y. Roh, H. D. Yoo, M. Choi, H. Kim, J. H. Kim, J. S. H. Lee, I. C. Park, G. Ryu, Y. Choi, Y. K. Choi, J. Goh, D. Kim, E. Kwon, J. Lee, I. Yu, A. Juodagalvis, J. Vaitkus, I. Ahmed, Z. A. Ibrahim, J. R. Komaragiri, M. A. B. Md Ali, F. Mohamad Idris, W. A. T. Wan Abdullah, M. N. Yusli, E. Casimiro Linares, H. Castilla-Valdez, E. De La Cruz-Burelo, I. Heredia-de La Cruz, A. Hernandez-Almada, R. Lopez-Fernandez, A. Sanchez-Hernandez, S. Carrillo Moreno, F. Vazquez Valencia, S. Carpinteyro, I. Pedraza, H. A. Salazar Ibarguen, A. Morelos Pineda, D. Krofcheck, P. H. Butler, S. Reucroft, A. Ahmad, M. Ahmad, Q. Hassan, H. R. Hoorani, W. A. Khan, T. Khurshid, M. Shoaib, H. Bialkowska, M. Bluj, B. Boimska, T. Frueboes, M. Górski, M. Kazana, K. Nawrocki, K. Romanowska-Rybinska, M. Szleper, P. Zalewski, G. Brona, K. Bunkowski, K. Doroba, A. Kalinowski, M. Konecki, J. Krolikowski, M. Misiura, M. Olszewski, M. Walczak, P. Bargassa, C. Beirão Da Cruz E Silva, A. Di Francesco, P. Faccioli, P. G. Ferreira Parracho, M. Gallinaro, N. Leonardo, L. Lloret Iglesias, F. Nguyen, J. Rodrigues Antunes, J. Seixas, O. Toldaiev, D. Vadruccio, J. Varela, P. Vischia, S. Afanasiev, P. Bunin, M. Gavrilenko, I. Golutvin, I. Gorbunov, A. Kamenev, V. Karjavin, V. Konoplyanikov, A. Lanev, A. Malakhov, V. Matveev, P. Moisenz, V. Palichik, V. Perelygin, S. Shmatov, S. Shulha, N. Skatchkov, V. Smirnov, A. Zarubin, V. Golovtsov, Y. Ivanov, V. Kim, E. Kuznetsova, P. Levchenko, V. Murzin, V. Oreshkin, I. Smirnov, V. Sulimov, L. Uvarov, S. Vavilov, A. Vorobyev, Yu. Andreev, A. Dermenev, S. Gninenko, N. Golubev, A. Karneyeu, M. Kirsanov, N. Krasnikov, A. Pashenkov, D. Tlisov, A. Toropin, V. Epshteyn, V. Gavrilov, N. Lychkovskaya, V. Popov, l. Pozdnyakov, G. Safronov, A. Spiridonov, E. Vlasov, A. Zhokin, A. Bylinkin, V. Andreev, M. Azarkin, I. Dremin, M. Kirakosyan, A. Leonidov, G. Mesyats, S. V. Rusakov, A. Vinogradov, A. Baskakov, A. Belyaev, E. Boos, M. Dubinin, L. Dudko, A. Ershov, A. Gribushin, V. Klyukhin, O. Kodolova, I. Lokhtin, I. Myagkov, S. Obraztsov, S. Petrushanko, V. Savrin, A Snigirev, I. Azhgirey, I. Bayshev, S. Bitioukov, V. Kachanov, A. Kalinin, D. Konstantinov, V. Krychkine, V. Petrov, R. Ryutin, A. Sobol, L. Tourtchanovitch, S. Troshin, N. Tyurin, A. Uzunian, A. Volkov, P. Adzic, M. Ekmedzic, J. Milosevic, V. Rekovic, J. Alcaraz Maestre, E. Calvo, M. Cerrada, M. Chamizo Llatas, N. Colino, B. De La Cruz, A. Delgado Peris, D. Domínguez Vázquez, A. Escalante Del Valle, C. Fernandez Bedoya, J. P. Fernández Ramos, J. Flix, M. C. Fouz, P. Garcia-Abia, O. Gonzalez Lopez, S. Goy Lopez, J. M. Hernandez, M. I. Josa, E. Navarro De Martino, A. Pérez-Calero Yzquierdo, J. Puerta Pelayo, A. Quintario Olmeda, I. Redondo, L. Romero, M. S. Soares, C. Albajar, J. F. de Trocóniz, M. Missiroli, D. Moran, H. Brun, J. Cuevas, J. Fernandez Menendez, S. Folgueras, I. Gonzalez Caballero, E. Palencia Cortezon, J. M. Vizan Garcia, I. J. Cabrillo, A. Calderon, J. R. Castiñeiras De Saa, P. De Castro Manzano, J. Duarte Campderros, M. Fernandez, G. Gomez, A. Graziano, A. Lopez Virto, J. Marco, R. Marco, C. Martinez Rivero, F. Matorras, F. J. Munoz Sanchez, J. Piedra Gomez, T. Rodrigo, A. Y. Rodríguez-Marrero, A. Ruiz-Jimeno, L. Scodellaro, I. Vila, R. Vilar Cortabitarte, D. Abbaneo, E. Auffray, G. Auzinger, M. Bachtis, P. Baillon, A. H. Ball, D. Barney, A. Benaglia, J. Bendavid, L. Benhabib, J. F. Benitez, G. M. Berruti, P. Bloch, A. Bocci, A. Bonato, C. Botta, H. Breuker, T. Camporesi, G. Cerminara, S. Colafranceschi, M. D’Alfonso, D. d’Enterria, A. Dabrowski, V. Daponte, A. David, M. De Gruttola, F. De Guio, A. De Roeck, S. De Visscher, E. Di Marco, M. Dobson, M. Dordevic, T. du Pree, N. Dupont, A. Elliott-Peisert, G. Franzoni, W. Funk, D. Gigi, K. Gill, D. Giordano, M. Girone, F. Glege, R. Guida, S. Gundacker, M. Guthoff, J. Hammer, M. Hansen, P. Harris, J. Hegeman, V. Innocente, P. Janot, H. Kirschenmann, M. J. Kortelainen, K. Kousouris, K. Krajczar, P. Lecoq, C. Lourenço, M. T. Lucchini, N. Magini, L. Malgeri, M. Mannelli, A. Martelli, L. Masetti, F. Meijers, S. Mersi, E. Meschi, F. Moortgat, S. Morovic, M. Mulders, M. V. Nemallapudi, H. Neugebauer, S. Orfanelli, L. Orsini, L. Pape, E. Perez, A. Petrilli, G. Petrucciani, A. Pfeiffer, D. Piparo, A. Racz, G. Rolandi, M. Rovere, M. Ruan, H. Sakulin, C. Schäfer, C. Schwick, A. Sharma, P. Silva, M. Simon, P. Sphicas, D. Spiga, J. Steggemann, B. Stieger, M. Stoye, Y. Takahashi, D. Treille, A. Triossi, A. Tsirou, G. I. Veres, N. Wardle, H. K. Wöhri, A. Zagozdzinska, W. D. Zeuner, W. Bertl, K. Deiters, W. Erdmann, R. Horisberger, Q. Ingram, H. C. Kaestli, D. Kotlinski, U. Langenegger, D. Renker, T. Rohe, F. Bachmair, L. Bäni, L. Bianchini, M. A. Buchmann, B. Casal, G. Dissertori, M. Dittmar, M. Donegà, M. Dünser, P. Eller, C. Grab, C. Heidegger, D. Hits, J. Hoss, G. Kasieczka, W. Lustermann, B. Mangano, A. C. Marini, M. Marionneau, P. Martinez Ruiz del Arbol, M. Masciovecchio, D. Meister, P. Musella, F. Nessi-Tedaldi, F. Pandolfi, J. Pata, F. Pauss, L. Perrozzi, M. Peruzzi, M. Quittnat, M. Rossini, A. Starodumov, M. Takahashi, V. R. Tavolaro, K. Theofilatos, R. Wallny, T. K. Aarrestad, C. Amsler, L. Caminada, M. F. Canelli, V. Chiochia, A. De Cosa, C. Galloni, A. Hinzmann, T. Hreus, B. Kilminster, C. Lange, J. Ngadiuba, D. Pinna, P. Robmann, F. J. Ronga, D. Salerno, S. Taroni, Y. Yang, M. Cardaci, K. H. Chen, T. H. Doan, C. Ferro, Sh. Jain, R. Khurana, M. Konyushikhin, C. M. Kuo, W. Lin, Y. J. Lu, R. Volpe, S. S. Yu, R. Bartek, P. Chang, Y. H. Chang, Y. W. Chang, Y. Chao, K. F. Chen, P. H. Chen, C. Dietz, F. Fiori, U. Grundler, W.-S. Hou, Y. Hsiung, Y. F. Liu, R.-S. Lu, M. Miñano Moya, E. Petrakou, J. F. Tsai, Y. M. Tzeng, B. Asavapibhop, K. Kovitanggoon, G. Singh, N. Srimanobhas, N. Suwonjandee, A. Adiguzel, M. N. Bakirci, C. Dozen, I. Dumanoglu, E. Eskut, S. Girgis, G. Gokbulut, Y. Guler, E. Gurpinar, I. Hos, E. E. Kangal, G. Onengut, K. Ozdemir, A. Polatoz, D. Sunar Cerci, M. Vergili, C. Zorbilmez, I. V. Akin, B. Bilin, S. Bilmis, B. Isildak, G. Karapinar, U. E. Surat, M. Yalvac, M. Zeyrek, E. A. Albayrak, E. Gülmez, M. Kaya, O. Kaya, T. Yetkin, K. Cankocak, S. Sen, F. I. Vardarlı, B. Grynyov, L. Levchuk, P. Sorokin, R. Aggleton, F. Ball, L. Beck, J. J. Brooke, E. Clement, D. Cussans, H. Flacher, J. Goldstein, M. Grimes, G. P. Heath, H. F. Heath, J. Jacob, L. Kreczko, C. Lucas, Z. Meng, D. M. Newbold, S. Paramesvaran, A. Poll, T. Sakuma, S. Seif El Nasr-storey, S. Senkin, D. Smith, V. J. Smith, K. W. Bell, A. Belyaev, C. Brew, R. M. Brown, D. J. A. Cockerill, J. A. Coughlan, K. Harder, S. Harper, E. Olaiya, D. Petyt, C. H. Shepherd-Themistocleous, A. Thea, L. Thomas, I. R. Tomalin, T. Williams, W. J. Womersley, S. D. Worm, M. Baber, R. Bainbridge, O. Buchmuller, A. Bundock, D. Burton, S. Casasso, M. Citron, D. Colling, L. Corpe, N. Cripps, P. Dauncey, G. Davies, A. De Wit, M. Della Negra, P. Dunne, A. Elwood, W. Ferguson, J. Fulcher, D. Futyan, G. Hall, G. Iles, G. Karapostoli, M. Kenzie, R. Lane, R. Lucas, L. Lyons, A.-M. Magnan, S. Malik, J. Nash, A. Nikitenko, J. Pela, M. Pesaresi, K. Petridis, D. M. Raymond, A. Richards, A. Rose, C. Seez, A. Tapper, K. Uchida, M. Vazquez Acosta, T. Virdee, S. C. Zenz, J. E. Cole, P. R. Hobson, A. Khan, P. Kyberd, D. Leggat, D. Leslie, I. D. Reid, P. Symonds, L. Teodorescu, M. Turner, A. Borzou, K. Call, J. Dittmann, K. Hatakeyama, A. Kasmi, H. Liu, N. Pastika, O. Charaf, S. I. Cooper, C. Henderson, P. Rumerio, A. Avetisyan, T. Bose, C. Fantasia, D. Gastler, P. Lawson, D. Rankin, C. Richardson, J. Rohlf, J. St. John, L. Sulak, D. Zou, J. Alimena, E. Berry, S. Bhattacharya, D. Cutts, N. Dhingra, A. Ferapontov, A. Garabedian, U. Heintz, E. Laird, G. Landsberg, Z. Mao, M. Narain, S. Sagir, T. Sinthuprasith, R. Breedon, G. Breto, M. Calderon De La Barca Sanchez, S. Chauhan, M. Chertok, J. Conway, R. Conway, P. T. Cox, R. Erbacher, M. Gardner, W. Ko, R. Lander, M. Mulhearn, D. Pellett, J. Pilot, F. Ricci-Tam, S. Shalhout, J. Smith, M. Squires, D. Stolp, M. Tripathi, S. Wilbur, R. Yohay, R. Cousins, P. Everaerts, C. Farrell, J. Hauser, M. Ignatenko, D. Saltzberg, E. Takasugi, V. Valuev, M. Weber, K. Burt, R. Clare, J. Ellison, J. W. Gary, G. Hanson, J. Heilman, M. Ivova Paneva, P. Jandir, E. Kennedy, F. Lacroix, O. R. Long, A. Luthra, M. Malberti, M. Olmedo Negrete, A. Shrinivas, H. Wei, S. Wimpenny, J. G. Branson, G. B. Cerati, S. Cittolin, R. T. D’Agnolo, A. Holzner, R. Kelley, D. Klein, J. Letts, I. Macneill, D. Olivito, S. Padhi, M. Pieri, M. Sani, V. Sharma, S. Simon, M. Tadel, A. Vartak, S. Wasserbaech, C. Welke, F. Würthwein, A. Yagil, G. Zevi Della Porta, D. Barge, J. Bradmiller-Feld, C. Campagnari, A. Dishaw, V. Dutta, K. Flowers, M. Franco Sevilla, P. Geffert, C. George, F. Golf, L. Gouskos, J. Gran, J. Incandela, C. Justus, N. Mccoll, S. D. Mullin, J. Richman, D. Stuart, I. Suarez, W. To, C. West, J. Yoo, D. Anderson, A. Apresyan, A. Bornheim, J. Bunn, Y. Chen, J. Duarte, A. Mott, H. B. Newman, C. Pena, M. Pierini, M. Spiropulu, J. R. Vlimant, S. Xie, R. Y. Zhu, V. Azzolini, A. Calamba, B. Carlson, T. Ferguson, Y. Iiyama, M. Paulini, J. Russ, M. Sun, H. Vogel, I. Vorobiev, J. P. Cumalat, W. T. Ford, A. Gaz, F. Jensen, A. Johnson, M. Krohn, T. Mulholland, U. Nauenberg, J. G. Smith, K. Stenson, S. R. Wagner, J. Alexander, A. Chatterjee, J. Chaves, J. Chu, S. Dittmer, N. Eggert, N. Mirman, G. Nicolas Kaufman, J. R. Patterson, A. Rinkevicius, A. Ryd, L. Skinnari, L. Soffi, W. Sun, S. M. Tan, W. D. Teo, J. Thom, J. Thompson, J. Tucker, Y. Weng, P. Wittich, S. Abdullin, M. Albrow, J. Anderson, G. Apollinari, L. A. T. Bauerdick, A. Beretvas, J. Berryhill, P. C. Bhat, G. Bolla, K. Burkett, J. N. Butler, H. W. K. Cheung, F. Chlebana, S. Cihangir, V. D. Elvira, I. Fisk, J. Freeman, E. Gottschalk, L. Gray, D. Green, S. Grünendahl, O. Gutsche, J. Hanlon, D. Hare, R. M. Harris, J. Hirschauer, B. Hooberman, Z. Hu, S. Jindariani, M. Johnson, U. Joshi, A. W. Jung, B. Klima, B. Kreis, S. Kwan, S. Lammel, J. Linacre, D. Lincoln, R. Lipton, T. Liu, R. Lopes De Sá, J. Lykken, K. Maeshima, J. M. Marraffino, V. I. Martinez Outschoorn, S. Maruyama, D. Mason, P. McBride, P. Merkel, K. Mishra, S. Mrenna, S. Nahn, C. Newman-Holmes, V. O’Dell, K. Pedro, O. Prokofyev, G. Rakness, E. Sexton-Kennedy, A. Soha, W. J. Spalding, L. Spiegel, L. Taylor, S. Tkaczyk, N. V. Tran, L. Uplegger, E. W. Vaandering, C. Vernieri, M. Verzocchi, R. Vidal, H. A. Weber, A. Whitbeck, F. Yang, H. Yin, D. Acosta, P. Avery, P. Bortignon, D. Bourilkov, A. Carnes, M. Carver, D. Curry, S. Das, G. P. Di Giovanni, R. D. Field, M. Fisher, I. K. Furic, J. Hugon, J. Konigsberg, A. Korytov, J. F. Low, P. Ma, K. Matchev, H. Mei, P. Milenovic, G. Mitselmakher, L. Muniz, D. Rank, R. Rossin, L. Shchutska, M. Snowball, D. Sperka, J. Wang, S. Wang, J. Yelton, S. Hewamanage, S. Linn, P. Markowitz, G. Martinez, J. L. Rodriguez, A. Ackert, J. R. Adams, T. Adams, A. Askew, J. Bochenek, B. Diamond, J. Haas, S. Hagopian, V. Hagopian, K. F. Johnson, A. Khatiwada, H. Prosper, V. Veeraraghavan, M. Weinberg, V. Bhopatkar, M. Hohlmann, H. Kalakhety, D. Mareskas-palcek, T. Roy, F. Yumiceva, M. R. Adams, L. Apanasevich, D. Berry, R. R. Betts, I. Bucinskaite, R. Cavanaugh, O. Evdokimov, L. Gauthier, C. E. Gerber, D. J. Hofman, P. Kurt, C. O’Brien, l. D. Sandoval Gonzalez, C. Silkworth, P. Turner, N. Varelas, Z. Wu, M. Zakaria, B. Bilki, W. Clarida, K. Dilsiz, S. Durgut, R. P. Gandrajula, M. Haytmyradov, V. Khristenko, J.-P. Merlo, H. Mermerkaya, A. Mestvirishvili, A. Moeller, J. Nachtman, H. Ogul, Y. Onel, F. Ozok, A. Penzo, C. Snyder, P. Tan, E. Tiras, J. Wetzel, K. Yi, I. Anderson, B. A. Barnett, B. Blumenfeld, D. Fehling, L. Feng, A. V. Gritsan, P. Maksimovic, C. Martin, K. Nash, M. Osherson, M. Swartz, M. Xiao, Y. Xin, P. Baringer, A. Bean, G. Benelli, C. Bruner, J. Gray, R. P. Kenny, D. Majumder, M. Malek, M. Murray, D. Noonan, S. Sanders, R. Stringer, Q. Wang, J. S. Wood, I. Chakaberia, A. Ivanov, K. Kaadze, S. Khalil, M. Makouski, Y. Maravin, A. Mohammadi, L. K. Saini, N. Skhirtladze, I. Svintradze, S. Toda, D. Lange, F. Rebassoo, D. Wright, C. Anelli, A. Baden, O. Baron, A. Belloni, B. Calvert, S. C. Eno, C. Ferraioli, J. A. Gomez, N. J. Hadley, S. Jabeen, R. G. Kellogg, T. Kolberg, J. Kunkle, Y. Lu, A. C. Mignerey, Y. H. Shin, A. Skuja, M. B. Tonjes, S. C. Tonwar, A. Apyan, R. Barbieri, A. Baty, K. Bierwagen, S. Brandt, W. Busza, I. A. Cali, Z. Demiragli, L. Di Matteo, G. Gomez Ceballos, M. Goncharov, D. Gulhan, G. M. Innocenti, M. Klute, D. Kovalskyi, Y. S. Lai, Y.-J. Lee, A. Levin, P. D. Luckey, C. Mcginn, C. Mironov, X. Niu, C. Paus, D. Ralph, C. Roland, G. Roland, J. Salfeld-Nebgen, G. S. F. Stephans, K. Sumorok, M. Varma, D. Velicanu, J. Veverka, J. Wang, T. W. Wang, B. Wyslouch, M. Yang, V. Zhukova, B. Dahmes, A. Finkel, A. Gude, P. Hansen, S. Kalafut, S. C. Kao, K. Klapoetke, Y. Kubota, Z. Lesko, J. Mans, S. Nourbakhsh, N. Ruckstuhl, R. Rusack, N. Tambe, J. Turkewitz, J. G. Acosta, S. Oliveros, E. Avdeeva, K. Bloom, S. Bose, D. R. Claes, A. Dominguez, C. Fangmeier, R. Gonzalez Suarez, R. Kamalieddin, J. Keller, D. Knowlton, I. Kravchenko, J. Lazo-Flores, F. Meier, J. Monroy, F. Ratnikov, J. E. Siado, G. R. Snow, M. Alyari, J. Dolen, J. George, A. Godshalk, I. Iashvili, J. Kaisen, A. Kharchilava, A. Kumar, S. Rappoccio, G. Alverson, E. Barberis, D. Baumgartel, M. Chasco, A. Hortiangtham, A. Massironi, D. M. Morse, D. Nash, T. Orimoto, R. Teixeira De Lima, D. Trocino, R.-J. Wang, D. Wood, J. Zhang, K. A. Hahn, A. Kubik, N. Mucia, N. Odell, B. Pollack, A. Pozdnyakov, M. Schmitt, S. Stoynev, K. Sung, M. Trovato, M. Velasco, S. Won, A. Brinkerhoff, N. Dev, M. Hildreth, C. Jessop, D. J. Karmgard, N. Kellams, K. Lannon, S. Lynch, N. Marinelli, F. Meng, C. Mueller, Y. Musienko, T. Pearson, M. Planer, A. Reinsvold, R. Ruchti, G. Smith, N. Valls, M. Wayne, M. Wolf, A. Woodard, L. Antonelli, J. Brinson, B. Bylsma, L. S. Durkin, S. Flowers, A. Hart, C. Hill, R. Hughes, K. Kotov, T. Y. Ling, B. Liu, W. Luo, D. Puigh, M. Rodenburg, B. L. Winer, H. W. Wulsin, O. Driga, P. Elmer, J. Hardenbrook, P. Hebda, S. A. Koay, P. Lujan, D. Marlow, T. Medvedeva, M. Mooney, J. Olsen, C. Palmer, P. Piroué, X. Quan, H. Saka, D. Stickland, C. Tully, J. S. Werner, A. Zuranski, S. Malik, V. E. Barnes, D. Benedetti, D. Bortoletto, L. Gutay, M. K. Jha, M. Jones, K. Jung, M. Kress, D. H. Miller, N. Neumeister, F. Primavera, B. C. Radburn-Smith, X. Shi, I. Shipsey, D. Silvers, J. Sun, A. Svyatkovskiy, F. Wang, W. Xie, L. Xu, J. Zablocki, N. Parashar, J. Stupak, A. Adair, B. Akgun, Z. Chen, K. M. Ecklund, F. J. M. Geurts, M. Guilbaud, W. Li, B. Michlin, M. Northup, B. P. Padley, R. Redjimi, J. Roberts, J. Rorie, Z. Tu, J. Zabel, B. Betchart, A. Bodek, P. de Barbaro, R. Demina, Y. Eshaq, T. Ferbel, M. Galanti, A. Garcia-Bellido, P. Goldenzweig, J. Han, A. Harel, O. Hindrichs, A. Khukhunaishvili, G. Petrillo, M. Verzetti, L. Demortier, S. Arora, A. Barker, J. P. Chou, C. Contreras-Campana, E. Contreras-Campana, D. Duggan, D. Ferencek, Y. Gershtein, R. Gray, E. Halkiadakis, D. Hidas, E. Hughes, S. Kaplan, R. Kunnawalkam Elayavalli, A. Lath, S. Panwalkar, M. Park, S. Salur, S. Schnetzer, D. Sheffield, S. Somalwar, R. Stone, S. Thomas, P. Thomassen, M. Walker, M. Foerster, G. Riley, K. Rose, S. Spanier, A. York, O. Bouhali, A. Castaneda Hernandez, M. Dalchenko, M. De Mattia, A. Delgado, S. Dildick, R. Eusebi, W. Flanagan, J. Gilmore, T. Kamon, V. Krutelyov, R. Montalvo, R. Mueller, I. Osipenkov, Y. Pakhotin, R. Patel, A. Perloff, J. Roe, A. Rose, A. Safonov, A. Tatarinov, K. A. Ulmer, N. Akchurin, C. Cowden, J. Damgov, C. Dragoiu, P. R. Dudero, J. Faulkner, S. Kunori, K. Lamichhane, S. W. Lee, T. Libeiro, S. Undleeb, I. Volobouev, E. Appelt, A. G. Delannoy, S. Greene, A. Gurrola, R. Janjam, W. Johns, C. Maguire, Y. Mao, A. Melo, P. Sheldon, B. Snook, S. Tuo, J. Velkovska, Q. Xu, M. W. Arenton, S. Boutle, B. Cox, B. Francis, J. Goodell, R. Hirosky, A. Ledovskoy, H. Li, C. Lin, C. Neu, E. Wolfe, J. Wood, F. Xia, C. Clarke, R. Harr, P. E. Karchin, C. Kottachchi Kankanamge Don, P. Lamichhane, J. Sturdy, D. A. Belknap, D. Carlsmith, M. Cepeda, A. Christian, S. Dasu, L. Dodd, S. Duric, E. Friis, B. Gomber, R. Hall-Wilton, M. Herndon, A. Hervé, P. Klabbers, A. Lanaro, A. Levine, K. Long, R. Loveless, A. Mohapatra, I. Ojalvo, T. Perry, G. A. Pierro, G. Polese, I. Ross, T. Ruggles, T. Sarangi, A. Savin, A. Sharma, N. Smith, W. H. Smith, D. Taylor, N. Woods, [Authorinst]The CMS Collaboration

**Affiliations:** 1 Yerevan Physics Institute, Yerevan, Armenia; 2 Institut für Hochenergiephysik der OeAW, Vienna, Austria; 3 National Centre for Particle and High Energy Physics, Minsk, Belarus; 4 Universiteit Antwerpen, Antwerp, Belgium; 5 Vrije Universiteit Brussel, Brussel, Belgium; 6 Université Libre de Bruxelles, Brussel, Belgium; 7 Ghent University, Ghent, Belgium; 8 Université Catholique de Louvain, Louvain-la-Neuve, Belgium; 9 Université de Mons, Mons, Belgium; 10 Centro Brasileiro de Pesquisas Fisicas, Rio de Janeiro, Brazil; 11 Universidade do Estado do Rio de Janeiro, Rio de Janeiro, Brazil; 12 Universidade Estadual Paulista, Universidade Federal do ABC, São Paulo, Brazil; 13 Institute for Nuclear Research and Nuclear Energy, Sofia, Bulgaria; 14 University of Sofia, Sofia, Bulgaria; 15 Institute of High Energy Physics, Beijing, China; 16 State Key Laboratory of Nuclear Physics and Technology, Peking University, Beijing, China; 17 Universidad de Los Andes, Bogotá, Colombia; 18 Faculty of Electrical Engineering, Mechanical Engineering and Naval Architecture, University of Split, Split, Croatia; 19 Faculty of Science, University of Split, Split, Croatia; 20 Institute Rudjer Boskovic, Zagreb, Croatia; 21 University of Cyprus, Nicosia, Cyprus; 22 Charles University, Prague, Czech Republic; 23 Academy of Scientific Research and Technology of the Arab Republic of Egypt, Egyptian Network of High Energy Physics, Cairo, Egypt; 24 National Institute of Chemical Physics and Biophysics, Tallinn, Estonia; 25 Department of Physics, University of Helsinki, Helsinki, Finland; 26 Helsinki Institute of Physics, Helsinki, Finland; 27 Lappeenranta University of Technology, Lappeenranta, Finland; 28 DSM/IRFU, CEA/Saclay, Gif-sur-Yvette, France; 29 Laboratoire Leprince-Ringuet, Ecole Polytechnique, IN2P3-CNRS, Palaiseau, France; 30 Institut Pluridisciplinaire Hubert Curien, Université de Strasbourg, Université de Haute Alsace Mulhouse, CNRS/IN2P3, Strasbourg, France; 31 Centre de Calcul de l’Institut National de Physique Nucleaire et de Physique des Particules, CNRS/IN2P3, Villeurbanne, France; 32 Institut de Physique Nucléaire de Lyon, Université de Lyon, Université Claude Bernard Lyon 1, CNRS-IN2P3, Villeurbanne, France; 33 Georgian Technical University, Tbilisi, Georgia; 34 Tbilisi State University, Tbilisi, Georgia; 35 I. Physikalisches Institut, RWTH Aachen University, Aachen, Germany; 36 III. Physikalisches Institut A, RWTH Aachen University, Aachen, Germany; 37 III. Physikalisches Institut B, RWTH Aachen University, Aachen, Germany; 38 Deutsches Elektronen-Synchrotron, Hamburg, Germany; 39 University of Hamburg, Hamburg, Germany; 40 Institut für Experimentelle Kernphysik, Karlsruhe, Germany; 41 Institute of Nuclear and Particle Physics (INPP), NCSR Demokritos, Aghia Paraskevi, Greece; 42 University of Athens, Athens, Greece; 43 University of Ioánnina, Ioannina, Greece; 44 Wigner Research Centre for Physics, Budapest, Hungary; 45 Institute of Nuclear Research ATOMKI, Debrecen, Hungary; 46 University of Debrecen, Debrecen, Hungary; 47 National Institute of Science Education and Research, Bhubaneswar, India; 48 Panjab University, Chandigarh, India; 49 University of Delhi, Delhi, India; 50 Saha Institute of Nuclear Physics, Kolkata, India; 51 Bhabha Atomic Research Centre, Mumbai, India; 52 Tata Institute of Fundamental Research, Mumbai, India; 53 Indian Institute of Science Education and Research (IISER), Pune, India; 54 Institute for Research in Fundamental Sciences (IPM), Tehran, Iran; 55 University College Dublin, Dublin, Ireland; 56 INFN Sezione di Bari, Università di Bari, Politecnico di Bari, Bari, Italy; 57 INFN Sezione di Bologna, Università di Bologna, Bologna, Italy; 58 INFN Sezione di Catania, Università di Catania, CSFNSM, Catania, Italy; 59 INFN Sezione di Firenze, Università di Firenze, Florence, Italy; 60 INFN Laboratori Nazionali di Frascati, Frascati, Italy; 61 INFN Sezione di Genova, Università di Genova, Genoa, Italy; 62 INFN Sezione di Milano-Bicocca, Università di Milano-Bicocca, Milan, Italy; 63 INFN Sezione di Napoli, Università di Napoli ‘Federico II’, Naples, Italy, Università della Basilicata, Potenza, Italy, Università G. Marconi, Rome, Italy; 64 INFN Sezione di Padova, Università di Padova, Padua, Italy, Università di Trento, Trento, Italy; 65 INFN Sezione di Pavia, Università di Pavia, Pavia, Italy; 66 INFN Sezione di Perugia, Università di Perugia, Perugia, Italy; 67 INFN Sezione di Pisa, Università di Pisa, Scuola Normale Superiore di Pisa, Pisa, Italy; 68 INFN Sezione di Roma, Università di Roma, Rome, Italy; 69 INFN Sezione di Torino, Università di Torino, Turin, Italy, Università del Piemonte Orientale, Novara, Italy; 70 INFN Sezione di Trieste, Università di Trieste, Trieste, Italy; 71 Kangwon National University, Chunchon, Korea; 72 Kyungpook National University, Daegu, Korea; 73 Chonbuk National University, Jeonju, Korea; 74 Institute for Universe and Elementary Particles, Chonnam National University, Kwangju, Korea; 75 Korea University, Seoul, Korea; 76 Seoul National University, Seoul, Korea; 77 University of Seoul, Seoul, Korea; 78 Sungkyunkwan University, Suwon, Korea; 79 Vilnius University, Vilnius, Lithuania; 80 National Centre for Particle Physics, Universiti Malaya, Kuala Lumpur, Malaysia; 81 Centro de Investigacion y de Estudios Avanzados del IPN, Mexico City, Mexico; 82 Universidad Iberoamericana, Mexico City, Mexico; 83 Benemerita Universidad Autonoma de Puebla, Puebla, Mexico; 84 Universidad Autónoma de San Luis Potosí, San Luis Potosí, Mexico; 85 University of Auckland, Auckland, New Zealand; 86 University of Canterbury, Christchurch, New Zealand; 87 National Centre for Physics, Quaid-I-Azam University, Islamabad, Pakistan; 88 National Centre for Nuclear Research, Swierk, Poland; 89 Institute of Experimental Physics, Faculty of Physics, University of Warsaw, Warsaw, Poland; 90 Laboratório de Instrumentação e Física Experimental de Partículas, Lisbon, Portugal; 91 Joint Institute for Nuclear Research, Dubna, Russia; 92 Petersburg Nuclear Physics Institute, Gatchina, St. Petersburg, Russia; 93 Institute for Nuclear Research, Moscow, Russia; 94 Institute for Theoretical and Experimental Physics, Moscow, Russia; 95 National Research Nuclear University ‘Moscow Engineering Physics Institute’ (MEPhI), Moscow, Russia; 96 P. N. Lebedev Physical Institute, Moscow, Russia; 97 Skobeltsyn Institute of Nuclear Physics, Lomonosov Moscow State University, Moscow, Russia; 98 State Research Center of Russian Federation, Institute for High Energy Physics, Protvino, Russia; 99 Faculty of Physics and Vinca Institute of Nuclear Sciences, University of Belgrade, Belgrade, Serbia; 100 Centro de Investigaciones Energéticas Medioambientales y Tecnológicas (CIEMAT), Madrid, Spain; 101 Universidad Autónoma de Madrid, Madrid, Spain; 102 Universidad de Oviedo, Oviedo, Spain; 103 Instituto de Física de Cantabria (IFCA), CSIC-Universidad de Cantabria, Santander, Spain; 104 CERN, European Organization for Nuclear Research, Geneva, Switzerland; 105 Paul Scherrer Institut, Villigen, Switzerland; 106 Institute for Particle Physics, ETH Zurich, Zurich, Switzerland; 107 Universität Zürich, Zurich, Switzerland; 108 National Central University, Chung-Li, Taiwan; 109 National Taiwan University (NTU), Taipei, Taiwan; 110 Department of Physics, Faculty of Science, Chulalongkorn University, Bangkok, Thailand; 111 Cukurova University, Adana, Turkey; 112 Physics Department, Middle East Technical University, Ankara, Turkey; 113 Bogazici University, Istanbul, Turkey; 114 Istanbul Technical University, Istanbul, Turkey; 115 Institute for Scintillation Materials of National Academy of Science of Ukraine, Kharkov, Ukraine; 116 National Scientific Center, Kharkov Institute of Physics and Technology, Kharkov, Ukraine; 117 University of Bristol, Bristol, UK; 118 Rutherford Appleton Laboratory, Didcot, UK; 119 Imperial College, London, UK; 120 Brunel University, Uxbridge, UK; 121 Baylor University, Waco, USA; 122 The University of Alabama, Tuscaloosa, USA; 123 Boston University, Boston, USA; 124 Brown University, Providence, USA; 125 University of California, Davis, Davis, USA; 126 University of California, Los Angeles, USA; 127 University of California, Riverside, Riverside, USA; 128 University of California, San Diego, La Jolla, USA; 129 University of California, Santa Barbara, Santa Barbara, USA; 130 California Institute of Technology, Pasadena, USA; 131 Carnegie Mellon University, Pittsburgh, USA; 132 University of Colorado Boulder, Boulder, USA; 133 Cornell University, Ithaca, USA; 134 Fermi National Accelerator Laboratory, Batavia, USA; 135 University of Florida, Gainesville, USA; 136 Florida International University, Miami, USA; 137 Florida State University, Tallahassee, USA; 138 Florida Institute of Technology, Melbourne, USA; 139 University of Illinois at Chicago (UIC), Chicago, USA; 140 The University of Iowa, Iowa City, USA; 141 Johns Hopkins University, Baltimore, USA; 142 The University of Kansas, Lawrence, USA; 143 Kansas State University, Manhattan, USA; 144 Lawrence Livermore National Laboratory, Livermore, USA; 145 University of Maryland, College Park, USA; 146 Massachusetts Institute of Technology, Cambridge, USA; 147 University of Minnesota, Minneapolis, USA; 148 University of Mississippi, Oxford, USA; 149 University of Nebraska-Lincoln, Lincoln, USA; 150 State University of New York at Buffalo, Buffalo, USA; 151 Northeastern University, Boston, USA; 152 Northwestern University, Evanston, USA; 153 University of Notre Dame, Notre Dame, USA; 154 The Ohio State University, Columbus, USA; 155 Princeton University, Princeton, USA; 156 University of Puerto Rico, Mayaguez, USA; 157 Purdue University, West Lafayette, USA; 158 Purdue University Calumet, Hammond, USA; 159 Rice University, Houston, USA; 160 University of Rochester, Rochester, USA; 161 The Rockefeller University, New York, USA; 162 Rutgers, The State University of New Jersey, Piscataway, USA; 163 University of Tennessee, Knoxville, USA; 164 Texas A&M University, College Station, USA; 165 Texas Tech University, Lubbock, USA; 166 Vanderbilt University, Nashville, USA; 167 University of Virginia, Charlottesville, USA; 168 Wayne State University, Detroit, USA; 169 University of Wisconsin, Madison, USA; 170 CERN, Geneva, Switzerland

## Abstract

A measurement of the W boson pair production cross section in proton-proton collisions at $\sqrt{s} = 8$ TeV is presented. The data collected with the CMS detector at the LHC correspond to an integrated luminosity of 19.4$\,\text {fb}^\text {-1}$. The ${{\mathrm{W} }^{+} }\mathrm{W}^{-}$ candidates are selected from events with two charged leptons, electrons or muons, and large missing transverse energy. The measured ${{\mathrm{W} }^{+} }\mathrm{W}^{-}$ cross section is $60.1\pm 0.9\,\text {(stat)} \pm 3.2\,\text {(exp)} \pm 3.1\,\text {(theo)} \pm 1.6\,\text {(lumi)} \text {\,pb} = 60.1\pm 4.8\text {\,pb}$, consistent with the standard model prediction. The ${{\mathrm{W} }^{+} }\mathrm{W}^{-}$ cross sections are also measured in two different fiducial phase space regions. The normalized differential cross section is measured as a function of kinematic variables of the final-state charged leptons and compared with several perturbative QCD predictions. Limits on anomalous gauge couplings associated with dimension-six operators are also given in the framework of an effective field theory. The corresponding 95 % confidence level intervals are $-5.7< c_{\mathrm {WWW}}/\Lambda ^2 < 5.9\,\mathrm{TeV}^{-2}$, $-11.4< c_{\mathrm {W}}/\Lambda ^2 < 5.4\,\mathrm{TeV}^{-2}$, $-29.2< c_{\mathrm {B}}/\Lambda ^2 < 23.9\,\mathrm{TeV}^{-2}$, in the HISZ basis.

## Introduction

The standard model (SM) description of electroweak and strong interactions can be tested through precision measurements of the ${{\mathrm{W} }^{+} }\mathrm{W}^{-}$ production cross section at hadron colliders. Among the massive vector boson pair production processes, ${{\mathrm{W} }^{+} }\mathrm{W}^{-}$ has the largest cross section.

At the CERN LHC, the SM vector boson pair production is dominated by the *s*-channel and *t*-channel quark-antiquark ($\mathrm{q} {\bar{\mathrm{q}}}$) annihilation diagrams, while the gluon-gluon ($\mathrm{g} \mathrm{g}$) diagrams contribute only 3 % to the total production cross section [1]. Previous cross section results on ${{\mathrm{W} }^{+} }\mathrm{W}^{-}$ production in pp collisions at a center-of-mass energy of $\sqrt{s} = 7\,\mathrm{TeV}$ are reported to be $52.4\pm 2.0\,\text {(stat)} \pm 4.5\,\text {(syst)} \pm 1.62\,\text {(lumi)} \text {\,pb}$ by CMS [2] and $54.4\pm 4.0\,\text {(stat)} \pm 3.9\,\text {(syst)} \pm 2.0\,\text {(lumi)} \text {\,pb}$ by ATLAS [3]. Results at $\sqrt{s} = 8\,\mathrm{TeV}$ are reported by CMS using 3.5$\,\text {fb}^\text {-1}$ of data [4] with a measured value of $69.9\pm 2.8\,\text {(stat)} \pm 5.6\,\text {(syst)} \pm 3.1\,\text {(lumi)} \text {\,pb}$. Also, a cross section measurement of ${{\mathrm{W} }^{+} }\mathrm{W}^{-}$ production in $\mathrm{p} {\bar{\mathrm{p}}}$ collisions at $\sqrt{s} = 1.96$ TeVhas been recently reported by CDF to be $14.0\pm 0.6\,\text {(stat)} {^{+1.2}_{-1.0}}\,\text {(syst)} \pm 0.8\,\text {(lumi)} \text {\,pb}$ [5]. Next-to-next-to-leading-order (NNLO) calculations for the ${{\mathrm{W} }^{+} }\mathrm{W}^{-}$ production in pp collisions at $\sqrt{s} = 8\mathrm{TeV}$ predict a cross section of $\sigma ^{\mathrm {NNLO}} (\mathrm{p} \mathrm{p} \rightarrow {{\mathrm{W} }^{+} }\mathrm{W}^{-} ) = 59.8^{+1.3}_{-1.1}\text {\,pb}$ [6]. In this ${{\mathrm{W} }^{+} }\mathrm{W}^{-}$ production calculation, processes involving the SM Higgs boson are not considered; it is estimated they would increase the total cross section by about 8 % for the Higgs boson mass of 125 GeV [7].

We measure the ${{\mathrm{W} }^{+} }\mathrm{W}^{-}$ production cross section in the fully leptonic decay channel by selecting events with two high transverse momentum ($p_{\mathrm {T}}$) electrons or muons (${\mathrm{e}}^{+} {\mathrm{e}}^{-}$, ${{\upmu }}^{+} {{\upmu }}^{-}$, ${\mathrm{e}}^{\pm } {\upmu }^{\pm }$), large missing transverse energy ($E_{\mathrm {T}}^{\text {miss}}$), and zero or one jet with high $p_{\mathrm {T}}$. We provide a more precise measurement than previous results [4] by using an improved analysis strategy and a larger data sample. The $p_{\mathrm {T}}$ of the ${{\mathrm{W} }^{+} }\mathrm{W}^{-}$ system receives large higher-order corrections because of the restriction on the number of jets. The dominant $\mathrm{q} {\bar{\mathrm{q}}}$ component of the signal production is modeled by resumming the large higher-order corrections to the ${{\mathrm{W} }^{+} }\mathrm{W}^{-}$ $p_{\mathrm {T}}$ distribution, thus improving the signal efficiency determination [8, 9]. The expected contribution, based on simulation, from Higgs-boson-mediated processes to the observed signal yield is subtracted. The data correspond to a total accumulated luminosity of 19.4$\,\text {fb}^\text {-1}$ at $\sqrt{s} = 8\mathrm{TeV}$.

Any deviation from the SM expectations in measured production rates or any possible change in certain kinematic distributions could provide evidence for effects from physics beyond the SM. New physics processes at high mass scales that alter the ${{\mathrm{W} }^{+} }\mathrm{W}^{-}$ production can be described by operators with mass dimensions larger than four in an effective field theory (EFT) framework. The higher-dimensional operators of the lowest order from purely electroweak processes have dimension six, and can generate anomalous trilinear gauge couplings (ATGC) [10]. Thus the measurement of the coupling constants provides an indirect search for new physics at mass scales not directly accessible by the LHC. Aside from the tests of the SM, ${{\mathrm{W} }^{+} }\mathrm{W}^{-}$ production represents an important background source in searches for new particles, and its precise measurement is therefore important in searches for new physics.

This paper is organized as follows. After a brief description of the CMS detector in Sect. 2 and of the data and simulated samples in Sect. 3, the event reconstruction and selection is detailed in Sect. 4. The background estimation is described in Sect. 5, followed by an estimate of the uncertainties in Sect. 6. Finally the results for the inclusive ${{\mathrm{W} }^{+} }\mathrm{W}^{-}$ production cross section and those in a given fiducial phase space are presented in Sect. 7. The normalized differential cross sections are shown in Sect. 8 and limits on ATGCs in Sect. 9. A summary is given in Sect. 10.

## The CMS detector

The CMS detector, described in detail in Ref. [11], is a multipurpose apparatus designed to study high $p_{\mathrm {T}}$ physics processes in proton-proton and heavy-ion collisions. A superconducting solenoid occupies the central region of the CMS detector, providing a magnetic field of 3.8 $\text {\,T}$ parallel to the beam direction. Charged-particle trajectories are measured by the silicon pixel and strip trackers, which cover a pseudorapidity region of $|\eta | < 2.5$. The crystal electromagnetic calorimeter (ECAL), and the brass/scintillator hadron calorimeter surround the tracking volume and cover $|\eta | < 3$. The steel/quartz-fiber Cherenkov hadron forward (HF) calorimeter extends the coverage to $|\eta | < 5$. The muon system consists of gas-ionization detectors embedded in the steel flux-return yoke outside the solenoid, and covers $|\eta | < 2.4$. The first level of the CMS trigger system (level 1), composed of custom hardware processors, is designed to select the most interesting events in less than 4 ${\upmu }$s, using information from the calorimeters and muon detectors. The level 1 output rate is up to 100$\text {\,kHz}$. The high-level trigger processor farm further reduces the event rate to a few hundred Hz before data storage.

## Data and simulated samples

The data samples used correspond to an integrated luminosity of 19.4$\,\text {fb}^\text {-1}$ at $\sqrt{s} = 8\,\mathrm{TeV}$. The luminosity is measured using data from the HF system and the pixel detector [12].

Events are selected with a combination of triggers that require one or two high-$p_{\mathrm {T}}$ electrons or muons with relatively tight lepton identification, some of them including also isolation. The single-electron trigger $p_{\mathrm {T}}$ threshold is 27 GeV whereas that for single muons is 24 GeV. For the dilepton triggers, the $p_{\mathrm {T}}$ thresholds of the leading and trailing leptons are 17 and 8 GeV, respectively. The trigger efficiency is measured in data using ${\mathrm{Z}} \rightarrow \ell ^+\ell ^-$ events recorded with a dedicated unbiased trigger [13]. The overall trigger efficiency is over 98 % for signal events from $\mathrm{q} {\bar{\mathrm{q}}}\rightarrow {{\mathrm{W} }^{+} }\mathrm{W}^{-}$ and $\mathrm{g} \mathrm{g} \rightarrow {{\mathrm{W} }^{+} }\mathrm{W}^{-}$ processes within our kinematic and selection region. The trigger efficiency is measured as a function of the lepton $p_{\mathrm {T}}$ and $\eta$. In addition, prescaled single-lepton triggers with $p_{\mathrm {T}}$ thresholds of 8 and 17GeVare used for some of the data-driven background estimations.

Several Monte Carlo (MC) event generators are used to simulate the signal and background processes. The MC samples are used to optimize the event selection, evaluate efficiencies and acceptances, and to estimate yields. For all MC samples, the response of the CMS detector is simulated using a detailed description of the detector based on the Geant4 package [14]. The simulated events are corrected for the trigger efficiency to match the data.

The $\mathrm{q} {\bar{\mathrm{q}}}\rightarrow {{\mathrm{W} }^{+} }\mathrm{W}^{-}$ component of the signal is generated with powheg 2.0 [15–19]. For comparison we also use $\mathrm{q} {\bar{\mathrm{q}}}\rightarrow {{\mathrm{W} }^{+} }\mathrm{W}^{-}$ signal samples generated with the MadGraph 5.1 [20] and mc@nlo 4.0 [21] event generators. The $\mathrm{g} \mathrm{g} \rightarrow {{\mathrm{W} }^{+} }\mathrm{W}^{-}$ signal component is generated using gg2ww 3.1 [22]. The sum of the $\mathrm{q} {\bar{\mathrm{q}}}\rightarrow {{\mathrm{W} }^{+} }\mathrm{W}^{-}$ and $\mathrm{g} \mathrm{g} \rightarrow {{\mathrm{W} }^{+} }\mathrm{W}^{-}$ components is normalized to the inclusive $\mathrm{p} \mathrm{p} \rightarrow {{\mathrm{W} }^{+} }\mathrm{W}^{-}$ cross section at NNLO [6] accuracy.

Background processes with top quarks, $\mathrm{t} {\bar{\mathrm{t}}}$ and $\mathrm{t} {\mathrm{W} }$, are generated with powheg. Higgs boson processes are considered part of the background. They represent about 8 % of the ${{\mathrm{W} }^{+} }\mathrm{W}^{-}$ cross section at $\sqrt{s} = 8\, \mathrm{TeV}$ [6], but have a smaller signal efficiency and represent only about 3 % of the expected signal yield. The gluon fusion and vector boson fusion modes are generated with powheg for a Higgs boson mass of 125GeVand normalized to the SM cross section [23]. The simulation of associated Higgs production uses the pythia 6.4 generator [24]. The interference between the Higgs boson production process and the ${{\mathrm{W} }^{+} }\mathrm{W}^{-}$ continuum process is found to be approximately 0.1 %; the interference is significant only with the $\mathrm{g} \mathrm{g} \rightarrow {{\mathrm{W} }^{+} }\mathrm{W}^{-}$ process. The ${\mathrm{W} }{\mathrm{Z}}$, ${\mathrm{Z}} {\mathrm{Z}}$, $\mathrm {V} \mathrm {V} \mathrm {V}$ ($\mathrm {V} = {\mathrm{W} }/{\mathrm{Z}}$), ${\mathrm{Z}}/\gamma ^*\rightarrow \ell ^+\ell ^-$, ${\mathrm{W} }\gamma ^{*}$, and W +jets processes are generated using MadGraph. All other background processes are generated using pythia 6.4.

The set of parton distribution functions (PDF) used is CTEQ6L [25] for leading order (LO) generators and CT10 [26] for next-to-leading-order (NLO) generators. All the event generators are interfaced to pythia 6.4 for the showering and hadronization of partons, except mc@nlo, which is interfaced to herwig 6 [27]. The tauola 2.7 package [28] is used in the simulation of $\tau$ decays to account for polarization effects.

In order to suppress the top quark background processes, the $\mathrm{p} \mathrm{p} \rightarrow {{\mathrm{W} }^{+} }\mathrm{W}^{-}$ cross section is measured with events that have no more than one high-$p_{\mathrm {T}}$ jet. The veto on high-$p_{\mathrm {T}}$ jets enhances the importance of logarithms of the jet $p_{\mathrm {T}}$, spoiling the convergence of fixed-order calculations and requiring the use of dedicated resummation techniques for an accurate prediction of differential distributions [8, 9]. The $p_{\mathrm {T}}$ of the jets produced in association with the ${{\mathrm{W} }^{+} }\mathrm{W}^{-}$ system is strongly correlated with the transverse momentum of the ${{\mathrm{W} }^{+} }\mathrm{W}^{-}$ system, $p_{\mathrm {T}} ^{{\mathrm{W} }{\mathrm{W} }}$, especially in the case where only one jet is produced. Thus, a precise modeling of the $p_{\mathrm {T}} ^{{\mathrm{W} }{\mathrm{W} }}$ distribution is necessary for the estimation of the jet veto efficiency. In Ref. [8], the logarithmic terms that contribute to the $p_{\mathrm {T}} ^{{\mathrm{W} }{\mathrm{W} }}$ distribution from $\mathrm{q} {\bar{\mathrm{q}}}\rightarrow {{\mathrm{W} }^{+} }\mathrm{W}^{-}$ are resummed to next-to-next-to-leading-logarithm precision using the technique of $p_{\mathrm {T}}$ resummation [29]. The simulated $\mathrm{q} {\bar{\mathrm{q}}}\rightarrow {{\mathrm{W} }^{+} }\mathrm{W}^{-}$ signal events are reweighted according to the ratio of the $p_{\mathrm {T}} ^{{\mathrm{W} }{\mathrm{W} }}$ distribution from the $p_{\mathrm {T}}$-resummed calculation and from powheg and pythia. An equivalent reweighting procedure is applied to mc@nlo and MadGraph MC generators. The weights have different effects for each MC generator; the change in the jet veto efficiency estimated with powheg is about 3 % whereas it is 1 % for mc@nlo and 4 % for MadGraph. We find good agreement between the jet veto efficiency estimated with powheg, mc@nlo, and MadGraph after the equivalent reweighting procedure is applied to these MC generators.

Additional simulated proton-proton interactions overlapping with the event of interest, denoted as pileup events, are added to the simulated samples to reproduce the vertex multiplicity distribution measured in data. The average value of pileup events per bunch crossing is approximately 21.

## Event reconstruction and selection

A particle-flow algorithm [30, 31] is used to reconstruct the observable particles in the event by an optimized combination of information from different subdetectors: clusters of energy deposits measured by the calorimeters and charged-particle tracks identified in the central tracking system and the muon detectors.

This analysis uses leptonic decays ${\mathrm{W} }\rightarrow \ell \nu$ ($\ell$ = $\mathrm{e}$, $\mu$), so the signal candidates consist of three final states: ${\mathrm{e}}^{+} {\mathrm{e}}^{-}$, ${{\upmu }}^{+} {{\upmu }}^{-}$, and ${\mathrm{e}}^{\pm } {\upmu }^{\pm }$. The signal candidates contain a small contribution from ${\mathrm{W} }\rightarrow \tau \nu _{\tau }$ processes with leptonic $\tau$ decays, even though the analysis is not optimized for this final state. The contribution of these leptonic $\tau$ decays to the final signal candidates is about 10 %.

For each signal event, two oppositely charged lepton candidates are required, both with $p_{\mathrm {T}} > 20 \,\mathrm{GeV}$ and with $|\eta | < 2.5 (2.4)$ for electrons (muons). Among the vertices identified in the event, the vertex with the largest $\sum p_{\mathrm {T}} ^2$, where the sum runs over all charged tracks associated with the vertex, is chosen as the primary one. The lepton candidates are required to be compatible with originating from this primary vertex.

Electron candidates are defined by a reconstructed particle track in the tracking detector pointing to a cluster of energy deposits in the ECAL. A multivariate approach to identify electrons is employed [32] combining several measured quantities describing the track quality, the ECAL cluster shape, and the compatibility of the measurements from the two subdetectors. The electron energy is measured primarily from the ECAL cluster energy deposit [33]. Muon candidates are identified by signals of particle tracks in the muon system that match a track reconstructed in the central tracking system. Minimum requirements on the number of hits and on the goodness-of-fit of the full track are imposed on the muon curvature measurement [34].

The signal electrons and muons are required to be isolated to distinguish them from the semileptonic decays of heavy quarks or the in-flight decays of hadrons. The $\Delta R = \sqrt{{(\Delta \eta )^2 + (\Delta \phi )^2}}$ variable is used to measure the separation between reconstructed objects in the detector, where $\phi$ is the azimuthal angle (in radians) of the trajectory of the object in the plane transverse to the direction of the proton beams, and therefore $\Delta \phi$ is the $\phi$ separation between objects; $\Delta \eta$ is the $\eta$ separation between objects. Isolation criteria are set based on the distribution of low-momentum particles in the ($\eta ,\phi$) region around the leptons. To remove the contribution from the overlapping pileup interactions in this isolation region, the charged particles included in the computation of the isolation variable are required to originate from the primary vertex. This track assignment to the primary vertex is fairly loose, and includes most of the tracks from b-quark or c-quark decays. The neutral component in the isolation $\Delta R$ cone is corrected by the average energy density deposited by those neutral particles that originated from additional interactions [35]. The correction is measured in a region of the detector away from the known hard scattering in a control sample.

Electron isolation is characterized by the ratio of the total $p_{\mathrm {T}}$ of the particles reconstructed in a $\Delta R =0.3$ cone around the electron, excluding the electron itself, to the $p_{\mathrm {T}}$ of the electron. Isolated electrons are selected by requiring this ratio to be below 10 %. For each muon candidate, the scalar sum of the $p_{\mathrm {T}}$ of all particles originating from the primary vertex is reconstructed in $\Delta R$ cones of several radii around the muon direction, excluding the contribution from the muon itself. This information is combined using a multivariate algorithm that exploits the differential energy deposition in the isolation region to discriminate between the signal of prompt muons and muons from hadron decays inside a jet. The exact threshold value depends on the muon $\eta$ and $p_{\mathrm {T}}$  [36].

Jets are reconstructed using the anti-$k_{\mathrm {T}}$ clustering algorithm [37] with a distance parameter of 0.5, as implemented in the FastJet package [38, 39]. The properties of the jets are modified by particles from pileup interactions. A combinatorial background arises from low-$p_{\mathrm {T}}$ jets from pileup interactions, which are clustered together with high-$p_{\mathrm {T}}$ jets from the primary interaction. A multivariate jet identifier is applied to separate jets from the primary interaction and those reconstructed from energy deposits associated with pileup interactions [40]. The discrimination is based on the differences in the jet shapes, on the relative multiplicity of charged and neutral components, and on the different $p_{\mathrm {T}}$ fractions carried by the hardest components. Tracks that come from pileup vertices are removed from the jet clustering. After jet identification, we apply a correction similar to the one applied for lepton isolation that accounts for the contributions from pileup. Jet energy corrections are applied as a function of the jet $p_{\mathrm {T}}$ and $\eta$ [41]. Studies of the jet multiplicity as a function of the number of vertices have been performed using Z+jets events, and no significant dependence was found. Since the jet energy resolution in data is somewhat worse than in simulation, the $p_{\mathrm {T}}$ values of simulated jets need to be spread randomly 5 % in order to describe data. After corrections the jets considered for the event categorization are required to have $p_{\mathrm {T}} >30 \,\mathrm{GeV}$ and $|\eta |<4.7$.

To reduce the background from top quark decays, events with two or more jets surviving the jet selection criteria are rejected. To further suppress the top quark background, two tagging techniques based on soft-muon and b-quark jet tagging are applied [42]. The first method vetoes events containing a soft muon from the semileptonic decay of the b quark. Soft-muon candidates are defined without isolation requirements and are required to have $p_{\mathrm {T}} > 3\,\mathrm{GeV}$. The second method uses b-jet tagging criteria based on the impact parameter of the constituent tracks. In particular, a track counting high-efficiency algorithm is used to veto those events with a jet tagged as b quark (t-tagged events). The combined reduction of the top quark background is about 50 % in the zero-jet category and above 80 % for events with one jet with $p_{\mathrm {T}} >30\,\mathrm{GeV}$.

The ${\vec {E}}_{\mathrm {T}}^{\text {miss}}$ variable is defined as the negative vector sum of the $p_{\mathrm {T}}$ of all reconstructed particles (charged or neutral) in the event. A *projected* $E_{\mathrm {T}}^{\text {miss}}$ variable [36] is defined as the component of ${\vec {E}}_{\mathrm {T}}^{\text {miss}}$ transverse to the nearest lepton if the lepton is situated within an azimuthal angular window of ${\pm } \pi /2$ from the ${\vec {E}}_{\mathrm {T}}^{\text {miss}}$ direction, otherwise the $|{\vec {E}}_{\mathrm {T}}^{\text {miss}} |$ is used. This variable is particularly effective in rejecting (1) ${\mathrm{Z}}/\gamma ^* \rightarrow {\uptau } ^+{\uptau } ^-$ events where ${\vec {E}}_{\mathrm {T}}^{\text {miss}}$ is preferentially aligned with leptons, and (2) ${\mathrm{Z}}/\gamma ^*\rightarrow \ell ^+\ell ^-$ events with poorly measured ${\vec {E}}_{\mathrm {T}}^{\text {miss}}$. Since the ${\vec {E}}_{\mathrm {T}}^{\text {miss}}$ resolution is degraded in a high pileup environment, two projected $E_{\mathrm {T}}^{\text {miss}}$ variables are defined: one constructed from all identified particles (proj. $E_{\mathrm {T}}^{\text {miss}}$), and another constructed from the charged particles attached to the primary vertex only (proj. track $E_{\mathrm {T}}^{\text {miss}}$). The minimum of the two is required to be above 20 GeV.

Events with dilepton masses below 12 GeVare also rejected to remove contributions from low-mass resonances. The same requirement is applied to the ${\mathrm{e}}^{\pm } {\upmu }^{\pm }$ final state to reject multijet and ${\mathrm{W} }\gamma$ background processes. Finally, the transverse momentum of the dilepton system $p_{\mathrm {T}} ^{\ell \ell }$ is required to be above 45 GeVin the ${\mathrm{e}}^{+} {\mathrm{e}}^{-}$ and ${{\upmu }}^{+} {{\upmu }}^{-}$ final states, and above 30 GeVin the ${\mathrm{e}}^{\pm } {\upmu }^{\pm }$ final state to reduce both the Drell–Yan background and events containing jets misidentified as leptons.

The Drell–Yan (DY) ${\mathrm{Z}}/\gamma ^*$ process is the largest source of same-flavor lepton pair production background because of its large production cross section and the finite resolution of the ${\vec {E}}_{\mathrm {T}}^{\text {miss}}$ measurement. In order to suppress this background, a few additional selection requirements are applied to the same-flavor final states. The component of the Drell–Yan production close to the Z boson peak is rejected by requiring the dilepton invariant mass $m_{\ell \ell }$ to be more than 15 GeVaway from the ${\mathrm{Z}}$ boson mass. To suppress the remaining off-peak contribution, a dedicated multivariate selection is used, combining $E_{\mathrm {T}}^{\text {miss}}$ variables, kinematic variables of the dilepton system, the transverse mass, the leading jet $p_{\mathrm {T}}$, and differences in azimuthal angle between the dilepton system and the leading jet and the ${\vec {E}}_{\mathrm {T}}^{\text {miss}}$ [36]. These selection requirements effectively reduce the Drell–Yan background by three orders of magnitude, while retaining more than 50 % of the signal.

To reduce the background from other diboson processes, such as ${\mathrm{W} }{\mathrm{Z}}$ and ${\mathrm{Z}} {\mathrm{Z}}$ production, any event that has an additional third lepton passing the identification and isolation requirements and having $p_{\mathrm {T}} > 10 \,\mathrm{GeV}$ is rejected. Any ${\mathrm{W} }\gamma$ production where the photon converts is suppressed by rejecting electrons consistent with a photon conversion [33].

A summary of the selection requirements for different- and same-flavor final states is shown in Table 1.

**Table 1:** Summary of the event selection for the different-flavor and same-flavor final states

Variable	Different-flavor	Same-flavor
Opposite-sign charge requirement	Applied	Applied
$p_{\mathrm {T}} ^{\ell }$ [$\text {GeV}$ ]	>20	>20
min(proj. $E_{\mathrm {T}}^{\text {miss}}$, proj. track $E_{\mathrm {T}}^{\text {miss}}$) [$\text {GeV}$ ]	>20	>20
DY MVA	–	>0.88 in zero-jet
		(>0.84 in one-jet)
$|m_{\ell \ell }- m_{{\mathrm{Z}}} |$ [$\text {GeV}$ ]	–	>15
$p_{\mathrm {T}} ^{\ell \ell }$ [$\text {GeV}$ ]	>30	>45
$m_{\ell \ell }$ [$\text {GeV}$ ]	>12	>12
Additional leptons ($p_{\mathrm {T}} ^{\ell } > 10\mathrm{GeV}$)	veto	veto
Top-quark veto	Applied	Applied
Number of reconstructed jets	<2	<2

## Estimation of backgrounds

**Fig. 1:**
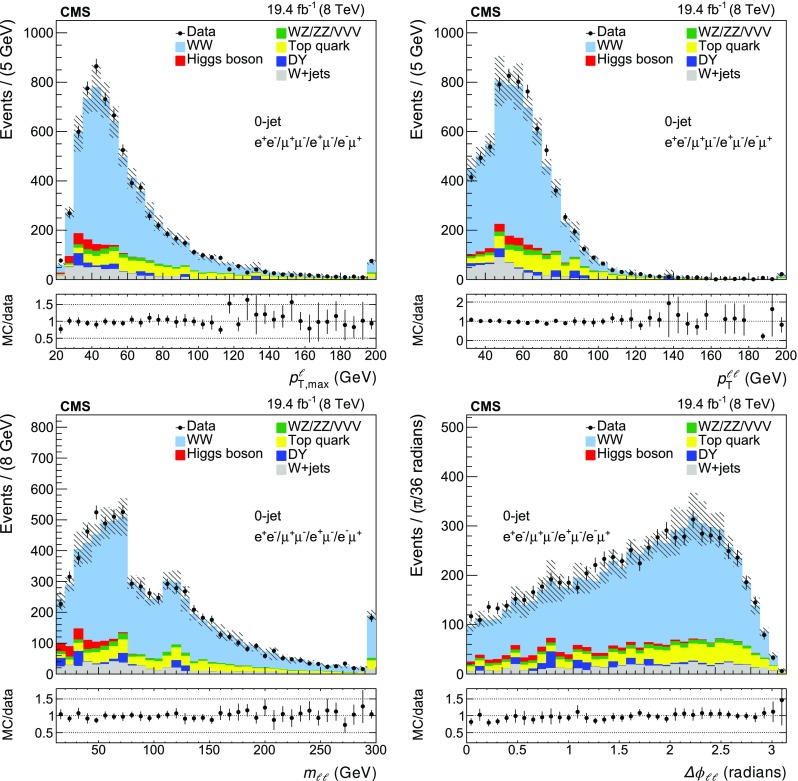
The data and MC distributions for the zero-jet category of the leading lepton $p_{\mathrm {T}}$ ($p_{{\mathrm {T}},\text { max}}^{\ell }$), the $p_{\mathrm {T}}$ of the dilepton system ($p_{\mathrm {T}} ^{\ell \ell }$), the dilepton invariant mass ($m_{\ell \ell }$) and the azimuthal angle between the two leptons ($\Delta \phi _{\ell \ell }$). The *hatched areas* represent the total systematic uncertainty in each bin. The *error bars* in the ratio plots are calculated considering the statistical uncertainty from the data sample and the systematic uncertainties in the background estimation and signal efficiencies. The last bin includes the overflow

**Fig. 2:**
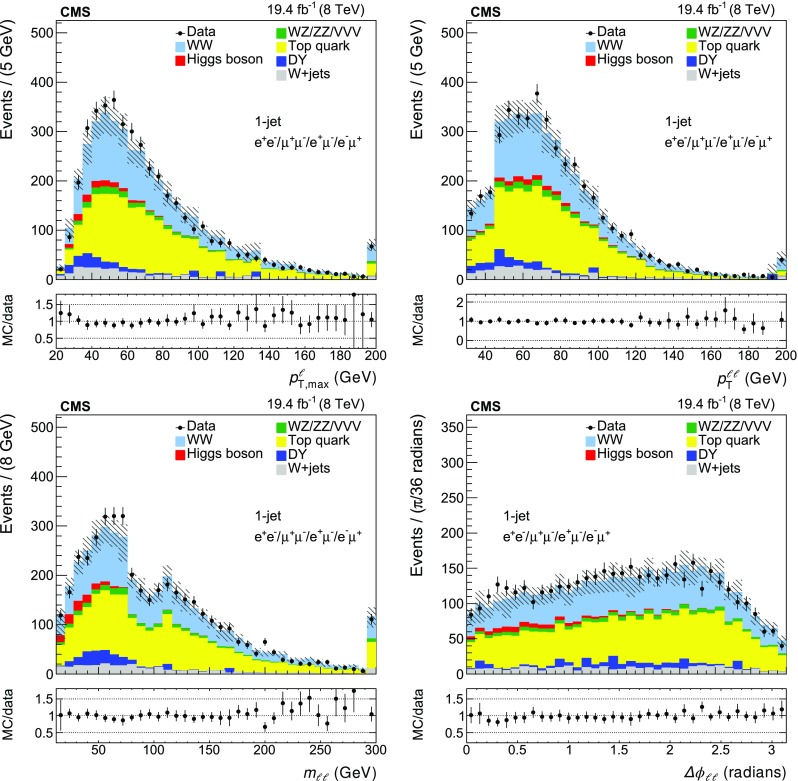
The data and MC distributions for the one-jet category of the leading lepton $p_{\mathrm {T}}$ ($p_{\mathrm{{T}},\text { max}}^{\ell }$), the $p_{\mathrm {T}}$ of the dilepton system ($p_{\mathrm {T}} ^{\ell \ell }$), the dilepton invariant mass ($m_{\ell \ell }$) and the azimuthal angle between the two leptons ($\Delta \phi _{\ell \ell }$). The *hatched areas* represent the total systematic uncertainty in each bin. The *error bars* in the ratio plots are calculated considering the statistical uncertainty from the data sample and the systematic uncertainties in the background estimation and signal efficiency. The last bin includes the overflow

A summary of the data, signal, and background yields for the different event categories is shown in Table 2. The distributions of the leading lepton $p_{\mathrm {T}}$ ($p_{{\mathrm {T}},\text { max}}^{\ell }$), the $p_{\mathrm {T}}$ of the dilepton system ($p_{\mathrm {T}} ^{\ell \ell }$), the dilepton invariant mass ($m_{\ell \ell }$) and the azimuthal angle between the two leptons ($\Delta \phi _{\ell \ell }$) are shown in Figs. 1 and  2 for the zero-jet and one-jet categories.

**Table 2:** Data, signal, and background yields for the four different event categories used for the $\mathrm{p} \mathrm{p} \rightarrow {{\mathrm{W} }^{+} }\mathrm{W}^{-}$ cross section measurement. The reported uncertainties include both statistical and systematic components as described in Sect. 6

Process	Zero-jet category		One-jet category	
	Different-flavor	Same-flavor	Different-flavor	Same-flavor
$\mathrm{q} {\bar{\mathrm{q}}}\rightarrow {{\mathrm{W} }^{+} }\mathrm{W}^{-}$	3516 ± 271	1390 ± 109	1113 ± 137	386 ± 49
$\mathrm{g} \mathrm{g} \rightarrow {{\mathrm{W} }^{+} }\mathrm{W}^{-}$	162 ± 50	91 ± 28	62 ± 19	27 ± 9
${{\mathrm{W} }^{+} }\mathrm{W}^{-}$	3678 ± 276	1481 ± 113	1174 ± 139	413 ± 50
${\mathrm{Z}} {\mathrm{Z}} +{\mathrm{W} }{\mathrm{Z}}$	84 ± 10	89 ± 11	86 ± 4	42 ± 2
$\mathrm {V} \mathrm {V} \mathrm {V}$	33 ± 17	17 ± 9	28 ± 14	14 ± 7
Top quark (${\mathrm {B}}_\text {t-tag}$)	522 ± 83	248 ± 26	1398 ± 156	562 ± 128
${\mathrm{Z}}/\gamma ^*\rightarrow \ell ^+\ell ^-$	38 ± 4	141 ± 63	136 ± 14	65 ± 33
${\mathrm{W} }\gamma ^{*}$	54 ± 22	12 ± 5	18 ± 8	3 ± 2
${\mathrm{W} }\gamma$	54 ± 20	20 ± 8	36 ± 14	9 ± 6
${\mathrm{W} }+\text {jets}(\mathrm{e})$	189 ± 68	46 ± 17	114 ± 41	16 ± 6
${\mathrm{W} }+{\text {jets}}{(\mu )}$	81 ± 40	19 ± 9	63 ± 30	17 ± 8
Higgs boson	125 ± 25	53 ± 11	75 ± 22	22 ± 7
Total bkg.	1179 ± 123	643 ± 73	1954 ± 168	749 ± 133
${{\mathrm{W} }^{+} }\mathrm{W}^{-}$ + total bkg.	4857 ± 302	2124 ± 134	3128 ± 217	1162 ± 142
Data	4847	2233	3114	1198

A combination of techniques is used to determine the contributions from backgrounds that remain after the ${{\mathrm{W} }^{+} }\mathrm{W}^{-}$ selection. A detailed description of these techniques can be found in Ref. [36]. The main background comes from top quark production, which is estimated from data. Instrumental backgrounds arising from misidentified (“nonprompt”) leptons in W +jets production and mismeasurement of ${\vec {E}}_{\mathrm {T}}^{\text {miss}}$ in ${\mathrm{Z}}/\gamma ^*$+jets events are also estimated from data. Other contributions from ${\mathrm{W} }\gamma$, ${\mathrm{W} }\gamma ^*$, and other subdominant diboson (${\mathrm{W} }{\mathrm{Z}}$ and ${\mathrm{Z}} {\mathrm{Z}}$) and triboson ($\mathrm {V} \mathrm {V} \mathrm {V}$) production processes are estimated partly from simulated samples.

A common scale factor is estimated for the $\mathrm{t} {\bar{\mathrm{t}}}$ and tW simulated samples. The top-quark background is suppressed using a top-tagging veto that eliminates visible top-quark decays. After the full event selection described in Table 1 but before the top-quark veto, the remaining top-quark background contribution ($\mathrm {B}_\text {t-tag}$) is estimated as: ${\mathrm {B}}_\text {t-tag} = {N}_\text {t-tag} \, (1-\epsilon _\text {t-tag})/\epsilon _\text {t-tag}$, where ${N}_\text {t-tag}$ is the number of t-tagged events before the top-quark veto, and $\epsilon _\text {t-tag}$ is the corresponding t-tagged efficiency. The number of t-tagged events (${N}_\text {t-tag}$) is determined in the signal data sample by counting the number of events passing the t-tagging requirements described in Sect. 4 and subtracting any remaining background on the basis of simulations or data, as described in the present section. The t-tagged efficiency ($\epsilon _\text {t-tag}$) is obtained from a measurement of the efficiency to tag a b-quark jet or soft muon in a top-enriched sample that consists of events with one (two) jet and exactly one b-tagged jet with $p_{\mathrm {T}} >30$ GeV, which isolates one b quark in a sample that is primarily $\mathrm{t} {\bar{\mathrm{t}}}$ or $\mathrm{t} {\mathrm{W} }$ events. Any remaining background is subtracted from the measured data in the top-enriched control sample. After excluding this b-tagged jet, the t-tagging efficiency is determined by counting the number of events that have an additional b-tagged jet or a soft muon. The measured efficiency is defined per b-quark decay and the value measured in the top-enriched sample is converted to a top-tagging efficiency in the signal region by taking into account the relative difference in the number of b-quark jets between the two samples after excluding the high-$p_{\mathrm {T}}$ b-tagged jet used to select events in the control sample. The conversion factor is calculated using the ratio of expected single-top $\mathrm{t} {\mathrm{W} }$ events to top-quark pair $\mathrm{t} {\bar{\mathrm{t}}}$ events in each region, and is done separately for the 0-jet and 1-jet categories as described in detail in Appendix D of Ref. [36]. We obtain efficiency values of 50–70 % in the signal samples. The main uncertainty comes from the statistical uncertainty in the control sample and from the systematic uncertainties related to the measurement of $\epsilon _\text {t-tag}$. The total uncertainty in $\mathrm{{B}}_\text {t-tag}$ amounts to about 13 % in the zero-jet category and 3 % in the one-jet category. The top background estimation method gives the estimate for the count of events in each of the four channels. This estimate is used to normalize the integral of the simulated distributions of $\mathrm{t} {\bar{\mathrm{t}}}$ and $\mathrm{t} {\mathrm{W} }$ backgrounds used in this paper.

The nonprompt lepton background occurs in W +jets and dijets production and originates from leptonic decays of heavy quarks, hadrons misidentified as leptons, and electrons from photon conversion. Most of it is suppressed by the identification and isolation requirements on electrons and muons described in Sect. 4. The remaining contribution is estimated directly from data from a sample enriched in nonprompt leptons. This sample is selected by choosing events with one lepton candidate that passes the standard lepton selection criteria, and another lepton candidate that fails the criteria, but passes a looser selection on impact parameter and isolation resulting in a sample of “pass-fail” lepton pairs. The yield in this sample is extrapolated to the signal region using the efficiencies for such loosely identified leptons to pass the standard lepton selection criteria.

The efficiency, $\epsilon _\text {pass}$, for a jet that satisfies the loose lepton requirements to pass the standard lepton selection is determined using an independent dijet sample. This independent dijet sample consists of events with one lepton candidate passing loose selection criteria and a recoiling jet, where contributions from ${\mathrm{W} }$+jets and $\mathrm{Z}$+jets events are suppressed by rejecting events with significant $E_{\mathrm {T}}^{\text {miss}}$ or with additional leptons. In order to study the composition of the nonprompt background, different dijet samples are defined by requiring different jet-$p_{\mathrm {T}}$ thresholds for the jet recoiling against the misidentified lepton. To ensure the measured efficiency is applicable to the signal region we compare the $p_{\mathrm {T}}$ spectrum of the jets in the dijet sample, and in the pass-fail sample from which the extrapolation is performed. The efficiency, parametrized as a function of $p_{\mathrm {T}}$ and $\eta$ of the lepton, is used to weight the events in the pass-fail sample by $\epsilon _\text {pass}/(1 - \epsilon _\text {pass})$ to obtain the estimated contribution from the nonprompt lepton background in the signal region. The systematic uncertainties from the determination of $\epsilon _\text {pass}$ dominate the overall uncertainty of this method. The systematic uncertainty is estimated by modifying the jet $p_{\mathrm {T}}$ threshold in the dijets sample, which modifies the jet sample composition, and from a closure test, where $\epsilon _\text {pass}$ is derived from simulated dijet events and applied to simulated background samples to predict the number of background events. The total uncertainty in $\epsilon _\text {pass}$ is of the order of 40 %, which includes the statistical uncertainty arising from the control sample size.

The ${\mathrm{Z}}/\gamma ^*\rightarrow \mathrm{e} \mathrm{e}/\mu \mu$ contribution, including ${\mathrm{Z}}/\gamma ^*\rightarrow \tau \tau$ leptonic decays, in the same-flavor final states outside of the Z boson mass window is obtained by normalizing the simulation. The normalization factor is defined by the ratio of the simulated to the observed number of events inside the ${\mathrm{Z}}$ boson mass window in data. The contribution of ${\mathrm{W} }{\mathrm{Z}}$ and ${\mathrm{Z}} {\mathrm{Z}}$ inside the ${\mathrm{Z}}$ boson mass window in data with neither lepton arising from a Z boson is subtracted before performing the normalization. This is done by counting the number of ${\mathrm{e}}^{\pm } {\upmu }^{\pm }$ events in the Z mass window, accounting for combinatorial effects and the relative detection efficiencies for electrons and muons. The contribution of ${\mathrm{W} }{\mathrm{Z}}$ and ${\mathrm{Z}} {\mathrm{Z}}$ processes in the Z mass window with leptons arising from different bosons, is also subtracted as estimated from simulation. The largest uncertainty in the estimate arises from the dependence of the extrapolation factor on $E_{\mathrm {T}}^{\text {miss}}$ and the multivariate Drell–Yan discriminant. The total uncertainty in the ${\mathrm{Z}}/\gamma ^*\rightarrow \ell ^+\ell ^-$ normalization is about 30 %, including both statistical and systematic components. The contribution of this background is also evaluated with an alternative method using $\gamma$ + jets events, which provides results consistent with the primary method. The ${\mathrm{Z}}/\gamma ^* \rightarrow {\uptau } ^+{\uptau } ^-$ background in the ${\mathrm{e}}^{\pm } {\upmu }^{\pm }$ channel is obtained from ${\mathrm{Z}}/\gamma ^*\rightarrow {{\upmu }}^{+} {{\upmu }}^{-}$ events selected in data, where the muons are replaced with simulated $\tau$ decays. The Drell–Yan event yield is rescaled to the observed yield using the inclusive sample of ${\mathrm{Z}}/\gamma ^*\rightarrow \ell ^+\ell ^-$ [43].

A data sample with three reconstructed leptons is selected in order to normalize the simulation used to estimate the ${\mathrm{W} }\gamma ^{*}$ background contribution coming from asymmetric $\gamma ^{*}$ decays where one lepton escapes detection [44]. The systematic uncertainty is estimated by comparing the normalization factor estimated in simulation in different regions. The uncertainty in the ${\mathrm{W} }\gamma ^{*}$ background estimate is of the order of 40 %.

Other backgrounds are estimated from simulation. The ${\mathrm{W} }\gamma$ background simulation is validated in data using the events passing all the selection requirements, except that the two leptons must have the same charge; this sample is dominated by W +jets and ${\mathrm{W} }\gamma$ events. Differences in the overall normalization are counted as a systematic uncertainty. The uncertainty in the ${\mathrm{W} }\gamma$ background estimate is about 30 %. Other minor backgrounds are ${\mathrm{W} }{\mathrm{Z}}$ and ${\mathrm{Z}} {\mathrm{Z}}$ diboson production where the two selected leptons come from different bosons.

## Signal efficiency and systematic uncertainties

The signal efficiency, which includes both detector geometrical acceptance and signal reconstruction and selection efficiency, is estimated using the $\mathrm{q} {\bar{\mathrm{q}}}\rightarrow {{\mathrm{W} }^{+} }\mathrm{W}^{-}$ and nonresonant (not through a Higgs resonance) $\mathrm{g} \mathrm{g} \rightarrow {{\mathrm{W} }^{+} }\mathrm{W}^{-}$ signal simulations described in Sect. 3. Signal events from ${\mathrm{W} }\rightarrow \tau \nu _{\tau }$ decays with $\tau$ leptons decaying into lower-energy electrons or muons are included in the signal efficiency. Residual discrepancies in the lepton reconstruction and identification efficiencies between data and simulation are corrected by applying data-to-simulation scale factors measured using ${\mathrm{Z}}/\gamma ^*\rightarrow \ell ^+\ell ^-$ events in the $\mathrm{Z}$ peak region [13] that are recorded with unbiased triggers. These factors depend on the lepton $p_{\mathrm {T}}$ and $\eta$ and are within 2 % (4 %) for electrons (muons). The uncertainty in the determination of the trigger efficiency leads to an uncertainty of about 1% in the expected signal yield. Any residual differences between the analysis lepton requirements with respect to the trigger selections are covered by the uncertainty in the trigger efficiency.

The experimental uncertainties in the lepton reconstruction and identification efficiency, momentum scale and resolution, $E_{\mathrm {T}}^{\text {miss}}$ modeling, and jet energy scale are applied to the reconstructed objects in simulated events by randomly spreading and scaling the relevant observables and propagating the effects to the kinematic variables used in the analysis. The distributions with varied detector response and resolution are used to estimate the change in the signal efficiency, whose value is taken as the associated systematic uncertainty. Uncertainties in lepton momentum scale and resolution are 0.5–4 % per lepton depending on the kinematics, and the effect on the yields at the analysis selection level is approximately 1 %. The uncertainties in the jet energy scale and resolution result in a 2–3 % uncertainty in the yields. The uncertainty in the resolution of the $E_{\mathrm {T}}^{\text {miss}}$ measurement is approximately 10 %, which is estimated from ${\mathrm{Z}}/\gamma ^*\rightarrow \ell ^+\ell ^-$ events with the same lepton selection as in the analysis. Randomly smearing the measured $E_{\mathrm {T}}^{\text {miss}}$ by one standard deviation of the resolution gives rise to 2 % variation in the estimation of signal yields after the full selection. A 2.6 % uncertainty is assigned to the integrated luminosity measurement [12].

The relative uncertainty in the signal acceptance from variations of the PDFs and the value of $\alpha _{s}$ in the simulated samples is estimated to be 1.3 % (0.8 %) for $\mathrm{q} {\bar{\mathrm{q}}}$ ($\mathrm{g} \mathrm{g}$) production, following the PDF4LHC prescription [23, 26, 45–48]. The effect of higher-order corrections in the $\mathrm{q} {\bar{\mathrm{q}}}\rightarrow {{\mathrm{W} }^{+} }\mathrm{W}^{-}$ signal acceptance is studied using the $p_{\mathrm {T}} ^{{\mathrm{W} }{\mathrm{W} }}$ reweighting procedure described in Sect. 3. Uncertainties are estimated by performing the reweighting while varying the resummation scale between half and twice the nominal value used in Ref. [8]. The reweighting functions with varied scales are then applied to simulated powheg events and used to calculate the variation in the signal acceptance. Uncertainties in the $\mathrm{q} {\bar{\mathrm{q}}}\rightarrow {{\mathrm{W} }^{+} }\mathrm{W}^{-}$ signal acceptance sensitive to the renormalization ($\mu _R$) and factorization ($\mu _F$) scales are estimated by varying both scales in the range ($\mu _0/2,2\mu _0$), with $\mu _0$ equal to the mass of the ${\mathrm{W} }$ boson, and setting $\mu _R = \mu _F$. The resummation scale uncertainty is found to be 2.8 % (6.9 %) for the zero-jet (one-jet) selection. The renormalization and factorization scales uncertainty is found to be 2.5 % (6.3 %) for the zero-jet (one-jet) selection. The systematic uncertainty associated with higher-order corrections to the $\mathrm{g} \mathrm{g} \rightarrow {{\mathrm{W} }^{+} }\mathrm{W}^{-}$ component of the signal is estimated by varying the renormalization and factorization scales and is found to be about 30 %.

The systematic uncertainties due to the underlying event and parton shower model are estimated by comparing samples with different MC event generators. In particular, the powheg MC generator interfaced with pythia for the parton shower and hadronization is compared to the mc@nlo generator interfaced with herwig for the parton shower and hadronization model. The systematic uncertainty is found to be 3.5 %.

The uncertainties in the background predictions are described in Sect. 5. The total uncertainty in the prediction of the top quark background is about 13 % (3 %) in the zero-jet (one-jet) categories, and about 36 % in the ${\mathrm{W} }+\text {jets}$ background prediction. The total uncertainty in the ${\mathrm{Z}}/\gamma ^*\rightarrow \ell ^+\ell ^-$ normalization is about 30 %, including both statistical and systematic contributions. The uncertainties in the yields of the ${\mathrm{Z}}/\gamma ^* \rightarrow {\uptau } ^+{\uptau } ^-$, ${\mathrm{W} }\gamma$, and ${\mathrm{W} }\gamma ^{*}$ background processes are 10, 30, and 40 %, respectively.

The theoretical uncertainties in the diboson cross sections are calculated by varying the renormalization and factorization scales using the mcfm 6.4 program [1]. The effects of variations in the PDFs and of the value of $\alpha _{s}$ on the predicted cross section are derived by following the same prescription as for the signal acceptance. Including the experimental uncertainties gives a systematic uncertainty of around 10 % for WZ and ZZ processes. In the case of ${\mathrm{W} }{\upgamma } ^{(*)}$ backgrounds, the variation in PDFs gives a systematic uncertainty of 4 %. A summary of the relative uncertainties in the ${{\mathrm{W} }^{+} }\mathrm{W}^{-}$ cross section measurement is given in Table 3, where the jet counting model uncertainty includes the renormalization and factorization scales, and underlying event uncertainties.

**Table 3:** Relative uncertainties in the ${{\mathrm{W} }^{+} }\mathrm{W}^{-}$ cross section measurement

Source	Uncertainty (%)
Statistical uncertainty	1.5
Lepton efficiency	3.8
Lepton momentum scale	0.5
Jet energy scale	1.7
$E_{\mathrm {T}}^{\text {miss}}$ resolution	0.7
$\mathrm{t} {\bar{\mathrm{t}}}$+$\mathrm{t} {\mathrm{W} }$ normalization	2.2
W +jets normalization	1.3
${\mathrm{Z}}/\gamma ^*\rightarrow \ell ^+\ell ^-$ normalization	0.6
${\mathrm{Z}}/\gamma ^* \rightarrow {\uptau } ^+{\uptau } ^-$ normalization	0.2
${\mathrm{W} }{\upgamma }$ normalization	0.3
${\mathrm{W} }{\upgamma } ^{*}$ normalization	0.4
$\mathrm {V} \mathrm {V}$ normalization	3.0
$\mathrm{H} \rightarrow {{\mathrm{W} }^{+} }\mathrm{W}^{-}$ normalization	0.8
Jet counting theory model	4.3
PDFs	1.2
MC statistical uncertainty	0.9
Integrated luminosity	2.6
Total uncertainty	7.9

## The ${{\mathrm{W} }^{+} }\mathrm{W}^{-}$ cross section measurement

The inclusive cross section is determined as

1 $$\begin{aligned} \sigma _{{{\mathrm{W} }^{+} }\mathrm{W}^{-}} = {\frac{N_\text {data}-N_\text {bkg} }{\mathcal {L} \, \epsilon \, \bigl ( 3 \, \mathcal {B}({\mathrm{W} }\rightarrow \ell {{\bar{\upnu }}}) \bigr )^2}}, \end{aligned}$$

where $N_\text {data}$ and $N_\text {bkg}$ are the total number of data and background events, $\epsilon$ is the signal efficiency, $\mathcal {L}$ is the integrated luminosity, and $\mathcal {B}({\mathrm{W} }\rightarrow \ell {{\bar{\upnu }}})$ is the branching fraction for a ${\mathrm{W} }$ boson decaying to each lepton family $\mathcal {B}({\mathrm{W} }\rightarrow \ell {{\bar{\upnu }}}) = (10.80 \pm 0.09)~\%$ [49].

The signal efficiency $\epsilon$ is evaluated as the fraction of the sum of $\mathrm{q} {\bar{\mathrm{q}}}\rightarrow {{\mathrm{W} }^{+} }\mathrm{W}^{-}$ and $\mathrm{g} \mathrm{g} \rightarrow {{\mathrm{W} }^{+} }\mathrm{W}^{-}$ generated events, with ${\mathrm{W} }\rightarrow \ell \nu$ ($\ell = \mathrm{e}$, $\mu$, $\tau$), accepted by the analysis selection. The efficiency estimated for each category is listed in Table 4. The reported statistical uncertainty in the efficiency originates from the limited size of the MC samples.

**Table 4:** Signal efficiency for the four event categories used in the $\mathrm{p} \mathrm{p} \rightarrow {{\mathrm{W} }^{+} }\mathrm{W}^{-}$ cross section measurement. The values reported are a product of the detector geometrical acceptance and the object reconstruction and event identification efficiency. The statistical uncertainty is from the limited size of the MC samples

Event category	Signal efficiency (%)
Zero-jet category	
Different-flavor	$3.02 \pm 0.02\,\text {(stat)} \pm 0.22\,\text {(syst)}$
Same-flavor	$1.21 \pm 0.01\,\text {(stat)} \pm 0.09\,\text {(syst)}$
One-jet category	
Different-flavor	$0.96 \pm 0.01\,\text {(stat)} \pm 0.11\,\text {(syst)}$
Same-flavor	$0.34 \pm 0.01\,\text {(stat)} \pm 0.04\,\text {(syst)}$

The ${{\mathrm{W} }^{+} }\mathrm{W}^{-}$ production cross section in $\mathrm{p} \mathrm{p}$ collision data at $\sqrt{s}=8\mathrm{TeV}$ is measured separately in events with same- and different-flavor leptons and in events with exclusively zero or one reconstructed and identified jet, as shown in Table 5. The number of events in each category, as shown in Table 2, is modeled as a Poisson random variable, whose mean value is the sum of the contributions from the processes under consideration. Systematic uncertainties are represented by individual nuisance parameters with log-normal distributions. The experimental and theoretical uncertainties in the event selection as well as the uncertainty on the integrated luminosity are reported separately. The theoretical component includes contributions from the jet counting theory model and PDFs as in Table 3. The measurement in the different flavor final state is consistent with that in the same flavor final state at the level of 1.5$\sigma$ after taking into account the statistical uncertainty and the uncorrelated systematic uncertainties.

**Table 5:** The ${{\mathrm{W} }^{+} }\mathrm{W}^{-}$ production cross section in each of the four event categories

Event category	${{\mathrm{W} }^{+} }\mathrm{W}^{-}$ production cross section (pb)
Zero-jet category	
Different-flavor	$59.7\pm 1.1\,\text {(stat)} \pm 3.3\,\text {(exp)} \pm 3.5\,\text {(theo)} \pm 1.6\,\text {(lumi)}$
Same-flavor	$64.3\pm 2.1\,\text {(stat)} \pm 4.6\,\text {(exp)} \pm 4.3\,\text {(theo)} \pm 1.7\,\text {(lumi)}$
One-jet category	
Different-flavor	$59.1\pm 2.8\,\text {(stat)} \pm 6.0\,\text {(exp)} \pm 6.2\,\text {(theo)} \pm 1.6\,\text {(lumi)}$
Same-flavor	$65.1\pm 5.5\,\text {(stat)} \pm 8.3\,\text {(exp)} \pm 8.0\,\text {(theo)} \pm 1.7\,\text {(lumi)}$

The four event categories are combined by performing a profile likelihood fit to the data following the statistical methodology described in Refs. [50–52]. The combined result is:

2 $$\begin{aligned} \sigma _{{{\mathrm{W} }^{+} }\mathrm{W}^{-}}= & {} 60.1\pm 0.9\,\text {(stat)} \pm 3.2\,\text {(exp)} \pm 3.1\,\text {(theo)} \nonumber \\&\pm 1.6\,\text {(lumi)} \text {\,pb} = 60.1\pm 4.8\text {\,pb}. \end{aligned}$$

The combined result shows good agreement with the NNLO theoretical prediction of $59.8^{+1.3}_{-1.1}\text {\,pb}$ [6]. The measurement precision is dominated by the result in the different-flavor zero-jet event category. The main source of systematic uncertainty comes from the modeling of the signal efficiency, especially the requirement on the number of reconstructed and identified jets.

We report the ${{\mathrm{W} }^{+} }\mathrm{W}^{-}$ production cross section in a fiducial region defined by a jet veto requirement in order to be less sensitive to theoretical uncertainties related to the modelling of the signal efficiency, especially those related to the requirement on the number of reconstructed and identified jets. When specifying the fiducial regions at generation level, jets are defined at particle level, before the detector effects, and clustered using the same anti-$k_{\mathrm {T}}$ algorithm with distance parameter of 0.5 as is used for collider data reconstruction. We measure the cross sections in a fiducial region defined by requiring no jets with $|\eta ^{\text {jet}} |<4.7$ and jet $p_{\mathrm {T}}$ above a series of thresholds. The results are summarized in Table 6 and compared with the predicted cross sections estimated with powheg. These results are consistent with the SM expectations.

**Table 6:** The ${{\mathrm{W} }^{+} }\mathrm{W}^{-}$ production cross section in fiducial regions defined by requiring no jets at particle level with jet $p_{\mathrm {T}}$ thresholds as listed

$p_{\mathrm {T}} ^{\text {jet}}$ ($\text {GeV}$)	$\sigma _{\text {zero-jet}}$ measured (pb)	$\sigma _{\text {zero-jet}}$ predicted (pb)
>20	$36.2\pm 0.6\,\text {(stat)} \pm 2.1\,\text {(exp)} \pm 1.1\,\text {(theo)} \pm 0.9\,\text {(lumi)}$	$36.7\pm 0.1\,\text {(stat)}$
>25	$40.8\pm 0.7\,\text {(stat)} \pm 2.3\,\text {(exp)} \pm 1.3\,\text {(theo)} \pm 1.1\,\text {(lumi)}$	$40.9\pm 0.1\,\text {(stat)}$
>30	$44.0\pm 0.7\,\text {(stat)} \pm 2.5\,\text {(exp)} \pm 1.4\,\text {(theo)} \pm 1.1\,\text {(lumi)}$	$43.9\pm 0.1\,\text {(stat)}$

The ${{\mathrm{W} }^{+} }\mathrm{W}^{-}$ cross section is also measured in the different-flavor zero-jet category, which is the most precise channel. The fiducial region is defined at generation level by requiring no jets with $|\eta ^{\text {jet}} |<4.7$ and a given maximum jet $p_{\mathrm {T}}$ for events with prompt leptons with $p_{\mathrm {T}} >20\,\mathrm{GeV}$ and $|\eta |<2.5$ before final-state radiation. In this case leptonic $\tau$ decays are not considered as part of the signal. The signal efficiency for this selection at generator level excluding $\tau$ lepton decays is 31.8 % for a jet $p_{\mathrm {T}}$ threshold of $30\,\mathrm{GeV}$. The measured cross sections are summarized in Table 7 and compared with the predicted cross sections estimated with powheg.

**Table 7:** The ${{\mathrm{W} }^{+} }\mathrm{W}^{-}$ production cross section in fiducial regions defined by requiring zero jets at particle level with varying jet $p_{\mathrm {T}}$ thresholds and requiring prompt leptons with $p_{\mathrm {T}} >20\mathrm{GeV}$ and $|\eta |<2.5$, before final-state radiation

$p_{\mathrm {T}} ^{\text {jet}}$ ($\text {GeV}$)	$\sigma _{{\text {zero-jet}},{\mathrm{W} }\rightarrow \ell \nu }$ measured (fb)	$\sigma _{\mathrm{{zero-jet}},{\mathrm{W} }\rightarrow \ell \nu }$ predicted (fb)
>20	$223\pm 4\,\text {(stat)} \pm 13\,\text {(exp)} \pm 7\,\text {(theo)} \pm 6\,\text {(lumi)}$	$228\pm 1\,\text {(stat)}$
>25	$253\pm 5\,\text {(stat)} \pm 14\,\text {(exp)} \pm 8\,\text {(theo)} \pm 7\,\text {(lumi)}$	$254\pm 1\,\text {(stat)}$
>30	$273\pm 5\,\text {(stat)} \pm 15\,\text {(exp)} \pm 9\,\text {(theo)} \pm 7\,\text {(lumi)}$	$274\pm 1\,\text {(stat)}$

Since both fiducial cross section measurements are restricted to the zero-jet category, most systematic uncertainties are calculated in the same way as in the inclusive analysis, except the underlying event, PDFs, and renormalization and factorization scales effects related to the ${{\mathrm{W} }^{+} }\mathrm{W}^{-}$ signal. In these cases the uncertainty is estimated as the largest difference among the three signal MC generators, powheg, MadGraph, and mc@nlo, for the fraction of reconstructed events outside the fiducial region and passing the full analysis selection. Fractionally, the theoretical uncertainty changes from 5 to 3 %.

## Normalized differential ${{\mathrm{W} }^{+} }\mathrm{W}^{-}$ cross section measurement

The normalized differential ${{\mathrm{W} }^{+} }\mathrm{W}^{-}$ cross section ${(1/\sigma )} \, {\mathrm{d}\sigma }/{\mathrm{d}X}$ is determined as a function of different *X* variables: the leading lepton $p_{{\mathrm {T}},\text { max}}^{\ell }$, the transverse momentum of the dilepton system $p_{\mathrm {T}} ^{\ell \ell }$, the invariant mass $m_{\ell \ell }$, and the angular separation in the transverse plane between the two leptons $\Delta \phi _{\ell \ell }$. The measurements are performed using unfolded distributions from events with zero jets and the ${\mathrm{e}}^{\pm } {\upmu }^{\pm }$ final state only. Leptonic $\tau$ decays are not considered as part of the signal.

The fiducial cross section is determined by the event yield in each bin after subtracting backgrounds. Each distribution is then corrected for event selection efficiencies and for detector resolution effects in order to be compared with predictions from event generators. The detector resolution corrections vary between 5 and 15 % depending on the variable and the bin. The correction procedure is based on unfolding techniques, as implemented in the RooUnfold toolkit [53], which provides both singular value decomposition (SVD) [54] and the iterative Bayesian [55] methods. Both algorithms use a response matrix that correlates the observable with and without detector effects. Regularization parameters are tuned to obtain results that are robust against numerical instabilities and statistical fluctuations. The unfolding is performed with the SVD method, and we cross-check the results with the iterative Bayesian method. We found a good agreement within uncertainties between both methods. The differential cross section is derived by dividing the corrected number of events by the integrated luminosity and by the bin width.

For each measured distribution, a response matrix is evaluated using $\mathrm{q} {\bar{\mathrm{q}}}\rightarrow {{\mathrm{W} }^{+} }\mathrm{W}^{-}$ events (generated with powheg) and $\mathrm{g} \mathrm{g} \rightarrow {{\mathrm{W} }^{+} }\mathrm{W}^{-}$, after full detector simulation. In order to minimize the model uncertainties due to unnecessary extrapolations of the measurement outside the experimentally well-described phase space region, the normalized differential cross section is determined in a phase space defined at the particle level by considering prompt leptons before final-state radiation, with $p_{\mathrm {T}} > 20\,\mathrm{GeV}$ and $|\eta | < 2.5$. Events with one or more jets with $p_{\mathrm {T}} >30$ GeVand $|\eta |<$ 4.7 are rejected.

The systematic uncertainties in each bin are assessed from the variations of the nominal cross section by repeating the full analysis for every systematic variation. The difference with respect to the nominal value is taken as the final systematic uncertainty for each bin and each measured observable. By using this method, the possible correlations of the systematic uncertainties between bins are taken into account. Those systematic uncertainties that are correlated across all bins of the measurement, and therefore mainly affect the normalization, cancel out at least partially in the normalized cross section. The uncertainty also includes the statistical error propagation through the unfolding method using the covariance matrix and the difference in the response matrix from MadGraph, powheg, and mc@nlo, the latter being almost negligible.

**Fig. 3:**
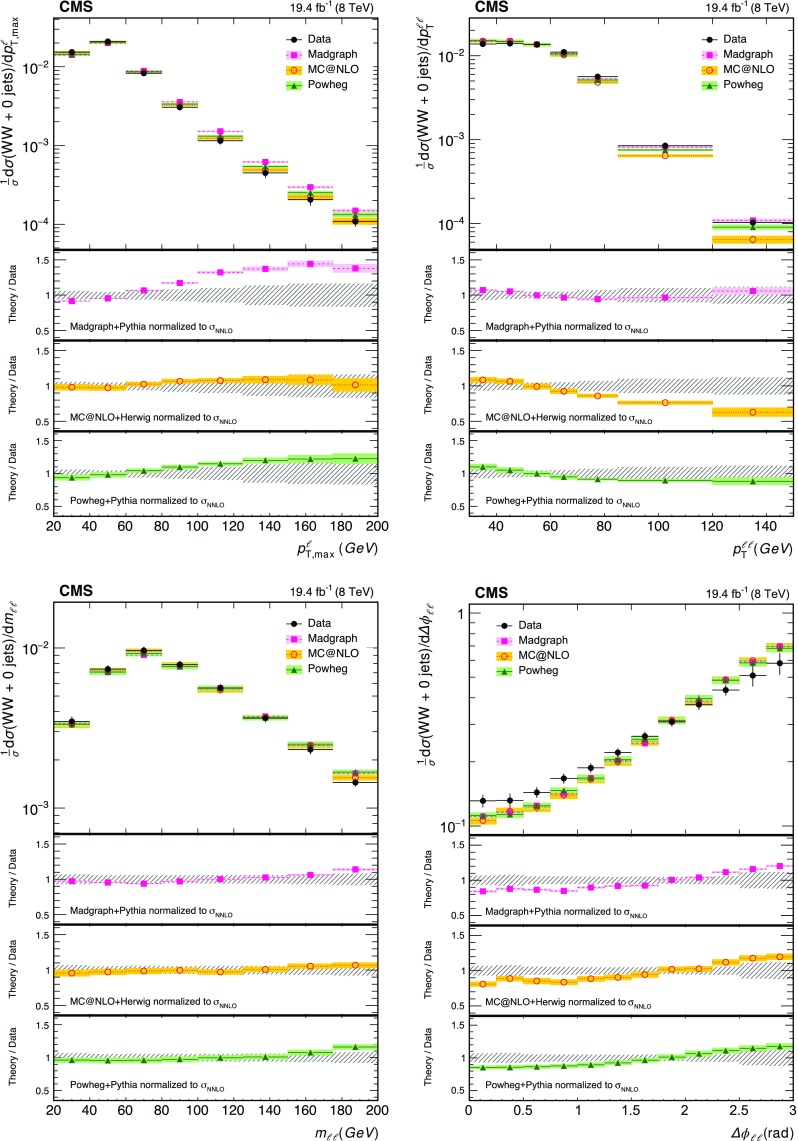
Normalized differential ${{\mathrm{W} }^{+} }\mathrm{W}^{-}$ cross section as a function of the leading lepton $p_{\mathrm {T}}$ ($p_{{\mathrm {T}},\text { max}}^{\ell }$) (*top left*), the transverse momentum of the dilepton system ($p_{\mathrm {T}} ^{\ell \ell }$) (*top right*), the invariant mass ($m_{\ell \ell }$) (*bottom left*) and the angular separation between leptons ($\Delta \phi _{\ell \ell }$) (*bottom right*). Both statistical and systematic uncertainties are included. The *hatched area* in the ratio plots corresponds to the relative error of the data in each bin. The measurement, including $\mathrm{g} \mathrm{g} \rightarrow {{\mathrm{W} }^{+} }\mathrm{W}^{-}$ is compared to predictions from MadGraph, powheg, and mc@nlo

Various differential cross sections in interesting kinematic variables are presented in Fig. 3. The measurements, including $\mathrm{g} \mathrm{g} \rightarrow {{\mathrm{W} }^{+} }\mathrm{W}^{-}$, are compared to the predictions from MadGraph, powheg, and mc@nlo, normalized to the recent QCD calculations up to approximate NNLO precision [6]. The predictions from MadGraph are shown with statistical uncertainties only. No single generator performs best for all the kinematic variables, although powheg does better than the others. Data and theory show a good agreement for the $m_{\ell \ell }$ and the $p_{\mathrm {T}} ^{\ell \ell }$ distributions, within uncertainties, except for the mc@nlo generator which predicts a softer $p_{\mathrm {T}} ^{\ell \ell }$ spectrum than observed. In case of the $p_{{\mathrm {T}},\text { max}}^{\ell }$ distribution, the MadGraph prediction shows an excess of events in the tail of the distribution compared to data, while powheg shows a reasonable agreement and mc@nlo shows a good agreement. We observe more significant differences in the shape of the $\Delta \phi _{\ell \ell }$ for all the three generators as compared to the data. Depending on the choice of MC generator, some of the differential cross sections show discrepancies up to 20 %, in extreme cases even up to 50 %, when comparing with a LO generator. These deviations are covered by the typical background uncertainties of Run 1 searches for physics beyond the SM. A better modelling of the WW background will be required to reduce the corresponding systematic uncertainties for Run 2, however.

## Limits on anomalous gauge couplings

Beyond-standard-model (BSM) physics effects in $\mathrm{p} \mathrm{p} \rightarrow {{\mathrm{W} }^{+} }\mathrm{W}^{-}$ can be described by a series of operators with mass dimensions larger than four in addition to the dimension-four operators in the SM Lagrangian. In the electroweak sector of the SM, in an EFT interpretation [10], the first higher-dimension operators made solely from electroweak vector fields and the Higgs doublet have mass dimension six. There are six different dimension-six operators that generate ATGCs. Three of them are C- and P-conserving while the others are not. In this analysis, we only consider models with C- and P-conserving operators. In the HISZ basis [56], these three operators are written as:

3 $$\begin{aligned} \frac{c_{\mathrm {WWW}}}{\Lambda ^2}\mathcal {O}_{\mathrm {WWW}}= & {} \frac{c_{\mathrm {WWW}}}{\Lambda ^2}\mathrm {Tr}[{\mathrm {W}}_{\mu \nu }{\mathrm {W}}^{\nu \rho }W_{\rho }^{\;\mu }],\nonumber \\ \frac{c_{\mathrm {W}}}{\Lambda ^2}\mathcal {O}_{\mathrm {W}}= & {} \frac{c_{\mathrm {W}}}{\Lambda ^2}(D^{\mu }\Phi )^{\dagger }{\mathrm {W}}_{\mu \nu }(D^{\nu }\Phi ),\nonumber \\ \frac{c_{\mathrm {B}}}{\Lambda ^2}\mathcal {O}_\mathrm{{B}}= & {} \frac{c_{\mathrm {B}}}{\Lambda ^2}(D^{\mu }\Phi )^{\dagger }{\mathrm {B}}_{\mu \nu }(D^{\nu }\Phi ). \end{aligned}$$

**Fig. 4:**
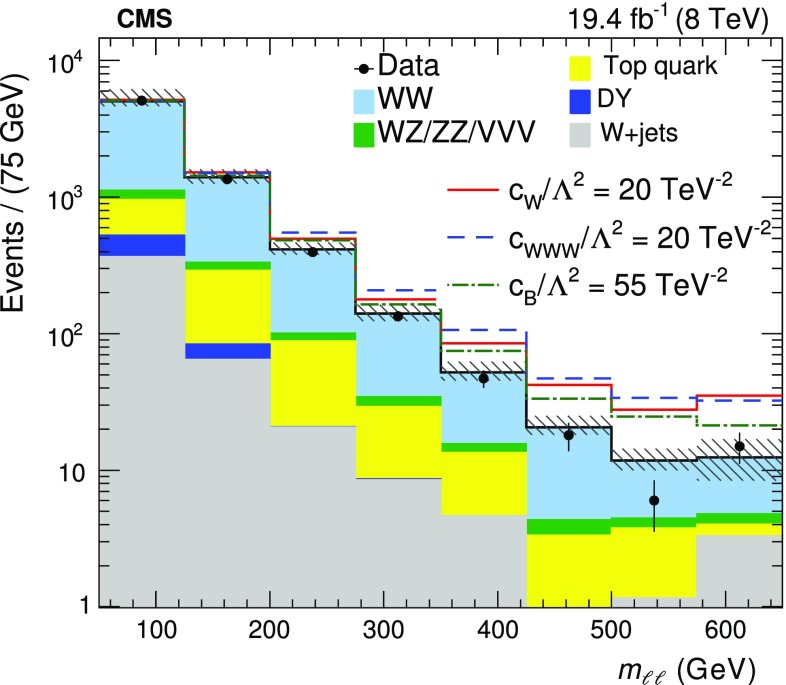
The $m_{\ell \ell }$ distribution with all SM backgrounds and $c_{\mathrm {W}}/\Lambda ^2 = 20\,\mathrm{TeV}^{-2}$, $c_{\mathrm {WWW}}/\Lambda ^2 = 20\,\mathrm{TeV}^{-2}$, and $c_{\mathrm {B}}/\Lambda ^2 = 55\,\mathrm{TeV}^{-2}$. The events are selected requiring no reconstructed jets with $p_{\mathrm {T}} > 30\,\mathrm{GeV}$ and $|\eta | < 4.7$. The last bin includes all events with $m_{\ell \ell } > 575\,\mathrm{GeV}$. The *hatched area* around the SM distribution is the total systematic uncertainty in each bin. The signal component is simulated with MadGraph and contains the $\mathrm{q} {\bar{\mathrm{q}}}\rightarrow {{\mathrm{W} }^{+} }\mathrm{W}^{-}$, the nonresonant $\mathrm{g} \mathrm{g} \rightarrow {{\mathrm{W} }^{+} }\mathrm{W}^{-}$, and the $\mathrm{g} \mathrm{g} \rightarrow \mathrm{H} \rightarrow {{\mathrm{W} }^{+} }\mathrm{W}^{-}$ components

**Fig. 5:**
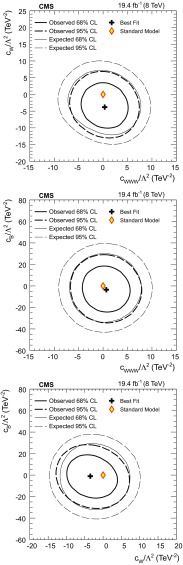
Two-dimensional observed (*thick lines*) and expected (*thin lines*) 68 and 95 % CL contours. The contours are obtained from profile log-likelihood comparisons to data assuming two nonzero coupling constants: $c_{\mathrm {WWW}}/\Lambda ^2 \times c_{\mathrm {W}}/\Lambda ^2$, $c_{\mathrm {WWW}}/\Lambda ^2 \times c_{\mathrm {B}}/\Lambda ^2$, and $c_{\mathrm {W}}/\Lambda ^2 \times c_{\mathrm {B}}/\Lambda ^2$. The *cross markers* indicate the best-fit values, and the *diamond markers* indicate the SM ones

The parameter $\Lambda$ is the mass scale that characterizes the coefficients of the higher-dimension operators, which can be regarded as the scale of new physics. The three operators in Eq. (3) generate both ATGC and Higgs boson anomalous couplings at tree level and modify the $\mathrm{p} \mathrm{p} \rightarrow {{\mathrm{W} }^{+} }\mathrm{W}^{-}$ cross section. In the absence of momentum-dependent form factors, the traditional LEP parametrization of ATGCs can be related to the values of the coupling constants of the dimension-six electroweak operators [10] as summarized in Eq. 4:

4 $$\begin{aligned} \delta (c_{\text {WWW}}/\Lambda ^2)= & {} \frac{2}{3g^2M_{{\mathrm{W} }^2}}\delta \lambda _{\gamma },\nonumber \\ \delta (c_{\text {W}}/\Lambda ^2)= & {} \frac{2}{M_{{\mathrm{Z}} ^2}}\delta g_1^{\mathrm{Z}},\nonumber \\ \delta (c_{\text {B}}/\Lambda ^2)= & {} 2\sqrt{\left( \frac{\delta \kappa _{\gamma }}{M_{{\mathrm{W} }^2}}\right) ^2+\left( \frac{\delta g_1^{\mathrm{Z}}}{M_{{\mathrm{Z}} ^2}}\right) ^2.} \end{aligned}$$

**Table 8:** Measured $c_{\mathrm {WWW}}/\Lambda ^2$, $c_{\mathrm {W}}/\Lambda ^2$, and $c_{\mathrm {B}}/\Lambda ^2$ coupling constants and their corresponding 95% CL intervals. Results are compared to the world average values, as explained in the text

Coupling constant	This result ($\text {TeV} ^{-2}$)	Its 95% CL interval ($\text {TeV} ^{-2}$)	World average ($\text {TeV} ^{-2}$)
$c_{\mathrm {WWW}}/\Lambda ^2$	$0.1_{-3.2}^{+3.2}$	[$-5.7, 5.9$]	$-5.5\pm 4.8$ (from $\lambda _{\gamma }$)
$c_{\mathrm {W}}/\Lambda ^2$	$-3.6_{-4.5}^{+5.0}$	[$-11.4, 5.4$]	$-3.9^{+3.9}_{-4.8}$ (from $g_1^{{\mathrm{Z}}}$)
$c_{\mathrm {B}}/\Lambda ^2$	$-3.2_{-14.5}^{+15.0}$	[$-29.2, 23.9$]	$-1.7^{+13.6}_{-13.9}$ (from $\kappa _{\gamma }$ and $g_1^{\mathrm{Z}}$)

The dataset selected for the ${{\mathrm{W} }^{+} }\mathrm{W}^{-}$ cross section measurement is used to bound $c_{{\mathrm {WWW}}}/\Lambda ^2$, $c_{\mathrm {W}}/\Lambda ^2$, and $c_{\mathrm {B}}/\Lambda ^2$. For this measurement, we require the events to have zero reconstructed and identified jets with $p_{\mathrm {T}} > 30\,\mathrm{GeV}$ and $|\eta | < 4.7$. We use the $m_{\ell \ell }$ distribution because it is robust against mismodeling of the transverse boost of the ${{\mathrm{W} }^{+} }\mathrm{W}^{-}$ system and is sensitive to the value of the coupling constants associated with the dimension-six operators. A binned Poisson log-likelihood comparing the data and simulated $m_{\ell \ell }$ distributions is computed. The template histograms representing various values of the ATGCs are prepared using ${{\mathrm{W} }^{+} }\mathrm{W}^{-}$ simulated events generated with MadGraph using a Lagrangian that contains the SM interaction terms and the three operators above. Thus, the simulation includes the pure SM contribution, the ATGC contribution, the Higgs boson anomalous coupling contribution, and the interference between the SM and ATGC contributions. The hard-scattering simulation includes up to one hard parton in the final state [57]. The detector response to the events is obtained using the detailed CMS detector simulation. The various background yields described in Sect. 5 are added to the $m_{\ell \ell }$ distribution from the simulated signal events. As an example of the templates, Fig. 4 shows the $m_{\ell \ell }$ distribution for one set of values of $c_{\mathrm {WWW}}/\Lambda ^2$, $c_{\mathrm {W}}/\Lambda ^2$, and $c_{\mathrm {B}}/\Lambda ^2$.

Templates of the $m_{\ell \ell }$ distribution are prepared for different hypothetical values of the coupling constants $c_{\mathrm {WWW}}/\Lambda ^2$, $c_{\mathrm {W}}/\Lambda ^2$, and $c_{\mathrm {B}}/\Lambda ^2$. We consider both the cases in which only one of the coupling constants has a nonzero value, and the cases in which two of them are varied simultaneously. The correlations between the measured coupling constants are not strong, so we do not consider the case in which the three coupling constants are allowed to vary simultaneously. Thus, the results presented here assume that the symmetries of the BSM theory would only allow either one or two of the dimension-six electroweak operators to contribute appreciably.

The expected number of events in each bin of the template histograms is interpolated using polynomial functions as a function of the coupling constants to create a continuous parametrization of the model. A profile likelihood fit to the data for each coupling-constant hypothesis is performed using the method described in Sect. 7.

Figure 5 shows the 2D likelihood profiles at 68 % and 95 % confidence levels (CL) for the three cases in which two coupling constants are allowed to vary. Using the templates prepared with a single non-zero coupling constant, we measure the values of $c_{\mathrm {WWW}}/\Lambda ^2$, $c_{\mathrm {W}}/\Lambda ^2$, and $c_{\mathrm {B}}/\Lambda ^2$ individually. The result of the 1D likelihood fit at 95 % CL intervals are given in Table 8.

In general, EFT predictions are valid if they maintain a separation between the scale of the momentum transfer in the process and the scale of new physics and if they preserve unitarity [58]. The first condition implies an upper bound on $|(c/\Lambda ^2)\hat{s} |$ of $(4\pi )^2 \approx 158$, although a specific new physics model may be more restrictive. The second condition requires an analysis of each operator, and sets the limits [59]: $|(c_{\text {WWW}}/\Lambda ^2)\hat{s} | < 85$, $|(c_{\text {W}}/\Lambda ^2)\hat{s} | < 205$, and $|(c_{\text {B}}/\Lambda ^2)\hat{s} | < 640$. For the experimental limits on the operator $\mathcal {O}_{\text {WWW}}$ given on Table 8, the most stringent constraint comes from the second condition and implies validity for $\sqrt{\hat{s}} < 3.8\,\mathrm{TeV}$. The operators $\mathcal {O}_{\text {W}}$ and $\mathcal {O}_{\text {B}}$ are constrained by the first condition to be valid for $\sqrt{\hat{s}} < 3.7\,\mathrm{TeV}$ and $\sqrt{\hat{s}} < 2.3\,\mathrm{TeV}$, respectively. In all three cases we expect all the data to have $\sqrt{\hat{s}}$ within the EFT range of validity. At the extreme hypothesis, for which the bounds are derived, only 3 % of the selected ${{\mathrm{W} }^{+} }\mathrm{W}^{-}$ events are expected to have $\sqrt{\hat{s}} > 2.3\,\mathrm{TeV}$. Within the limits of this interpretation, no evidence for anomalous WWZ and WW$\gamma$ triple gauge-boson couplings is found. Our results are compared to the world average values expressed in terms of $\lambda _{\gamma }$, $g_1^{\mathrm{Z}}$ and $\kappa _{\gamma }$ couplings. These world average values are driven by the LEP results [49, 60]. The conversion of the world average values from $\lambda _{\gamma }$, $g_1^{\mathrm{Z}}$ and $\kappa _{\gamma }$ couplings to the EFT formalism is done using the results from Ref. [10] and ignoring correlations as summarized in Eq. 4. These results represent an improvement in the measurement of $c_{\text {WWW}}/\Lambda ^{2}$.

## Summary

This paper reports a measurement of the ${{\mathrm{W} }^{+} }\mathrm{W}^{-}$ cross section in pp collisions at a center of mass energy of 8 TeV, using an integrated luminosity of $\mathcal {L} = 19.4\pm 0.5{\,\text {fb}^\text {-1}}$. The measured ${{\mathrm{W} }^{+} }\mathrm{W}^{-}$ cross section is $60.1 \pm 0.9\,\text {(stat)} \pm 3.2\,\text {(exp)} \pm 3.1\,\text {(theo)} \pm 1.6\,\text {(lumi)} \text {\,pb}$ = $60.1\pm 4.8\text {\,pb}$, consistent with the NNLO theoretical prediction $\sigma ^{\mathrm {NNLO}} (\mathrm{p} \mathrm{p} \rightarrow {{\mathrm{W} }^{+} }\mathrm{W}^{-} ) = 59.8^{+1.3}_{-1.1}\text {\,pb}$. We also report results on the normalized differential cross section measured as a function of kinematic variables of the final-state charged leptons and compared with several predictions from perturbative QCD calculations. Data and theory show a good agreement for the $m_{\ell \ell }$ and the $p_{\mathrm {T}} ^{\ell \ell }$ distributions within uncertainties, but the mc@nlo generator predicts a softer $p_{\mathrm {T}} ^{\ell \ell }$ spectrum compared with the data events. In case of the $p_{{\mathrm {T}},\text { max}}^{\ell }$ distribution, the MadGraph prediction shows an excess of events in the tail of the distribution compared to data, while powheg shows a reasonable agreement and mc@nlo shows a good agreement. We also observed differences in the shape of the $\Delta \phi _{\ell \ell }$ for the three generators compared to the data. No evidence for anomalous WWZ and WW$\gamma$ triple gauge-boson couplings is found, and limits on their magnitudes are set. These new limits are comparable to the current world average, and represent an improvement in the measurement of the coupling constant $c_{\mathrm {WWW}}/\Lambda ^{2}$.
